# *In vivo* assessment of brain microstructure in patients with Huntington’s disease using quantitative MRI

**DOI:** 10.1007/s00415-026-14244-8

**Published:** 2026-11-07

**Authors:** Marta M. Pokotylo, Joke-Lina Aßmann, Maximilian G. Ködderitzsch Mertins, Julia Henkel, Jan Uter, Leon van Well, Alexander Münchau, Sebastian Loens, Martin Göttlich, Norbert Brüggemann, Jannik Prasuhn

**Affiliations:** 1 Department of Neurology, Section for Movement Disorders, University of Luebeck, and University Hospital Schleswig-Holstein, Campus Luebeck, Ratzeburger Allee 160, 23562 Luebeck, Germany; 2 Center for Brain, Behaviour, and Metabolism, University of Luebeck, Luebeck, Germany; 3 Institute of Systems Motor Science, University of Luebeck, Luebeck, Germany; 4 Center for Rare Diseases, University of Luebeck, Luebeck, Germany; 5 Institute of Medical Psychology, University of Luebeck, Luebeck, Germany; 6 Institute of Neurogenetics, University of Luebeck, Luebeck, Germany; 7 Department of Neurology, Johns Hopkins University School of Medicine, Baltimore, MD USA; 8 F.M. Kirby Research Center for Functional Brain Imaging, Kennedy Krieger Institute, Baltimore, MD USA

**Keywords:** Multiparametric mapping (MPM), Huntington’s disease (HD), Quantitative MRI, In vivo histology, Basal ganglia

## Abstract

**Objectives:**

Using multiparametric mapping (MPM), we assessed the integrity of cortical and subcortical regions across different stages of Huntington’s disease (HD), relating the derived findings to genetic modifiers and clinical measures.

**Methods:**

Sixteen premanifest patients with HD (PwHD_pre_), ten manifesting PwHD (PwHD_man_), and forty-two healthy controls (HC) underwent clinical examinations and a 3 T imaging. We derived quantitative MPM maps to perform voxel-based quantification (VBQ) and voxel-based morphometry (VBM). We assessed group differences by combining PwHD_pre_ and PwHD_man_ (PwHD_total_) and by comparing PwHD_pre_ and PwHD_man_ with age- and sex-matched HC. Using correlation analysis, we investigated the link between MPM-derived findings, CAG repeat length, and clinical scores.

**Results:**

PwHD_man_ presented with reductions in all four quantitative MPM maps across the bilateral caudate nucleus and the frontal, parietal, temporal, and occipital gyri. We observed spatial overlap between VBQ- and VBM-derived findings, demonstrating widespread volume loss in PwHD_man_. PwHD_pre_ showed reductions in certain quantitative maps within the frontal and temporal regions. Reduced MPM values correlated with clinical scores of HD severity. Interpretation. The observed reductions across four MPM modalities in PwHD_man_ are consistent with demyelination and neuronal loss, as supported by widespread volume loss. The reductions observed in PwHD_pre_ indicate early demyelination and inflammation. In this study, we demonstrated the utility and clinical relevance of MPM for detecting microstructural alterations across different stages of HD. MPM can serve as a non-invasive biomarker of neurodegeneration in HD, providing insight into the pathological events underlying the observed neuronal loss.

**Supplementary Information:**

The online version contains supplementary material available at https://doi.org/10.1007/s00415-026-14244-8.

## Introduction

Huntington’s disease (HD) is an autosomal-dominant neurodegenerative disease caused by an expanded CAG repeat in the *huntingtin* (*HTT*) gene, with longer repeat lengths inversely correlating with earlier onset and greater disease severity [1, 2]. Although the causes of HD are well characterized, no disease-modifying treatments have been approved and only symptomatic strategies are available [3]. Furthermore, patients with HD (PwHD) exhibit highly heterogeneous symptomatology, spanning across motor, cognitive, and psychiatric domains, with emerging studies demonstrating symptom worsening with disease progression. Additionally, PwHD subgroups experience a more pronounced decline in specific domains, for reasons not yet understood [4, 5]. Such heterogeneous clinical presentation and disease progression, along with variable responses to symptomatic treatment, demonstrate the need for improved biomarkers to enhance patient stratification and monitoring.

Magnetic resonance imaging (MRI) is the most widely used neuroimaging tool for assessing basal ganglia (BG), a hallmark of HD pathology that correlates with motor symptom severity and disease duration [5, 6]. However, MRI is limited to detecting macroscopic structural changes and falls short of probing underlying molecular and pathogenic mechanisms [7]. Quantitative MRI (qMRI), such as multiparametric mapping (MPM), offers a more sensitive alternative by assessing qualitative measures of tissue biophysical properties and enabling evaluation of brain microstructural and cellular-level composition [8]. By modeling signals from multi-contrast gradient-echo acquisitions, MPM quantifies the longitudinal relaxation rate (R1), effective transverse relaxation rate (R2*), proton density (PD), and magnetization transfer saturation (MTsat) [9, 10]. As R1 and MTsat are sensitive to myelin, R2* to iron, and PD to the MR-visible mobile proton pool and tissue composition, MPM enables in vivo assessment of these tissue characteristics and the underlying pathogenic processes, yielding insights previously obtainable only with histology. Because histology assesses the end-stage of brain microstructure, its clinical relevance is limited, making MPM a better clinical alternative [8].

Despite its strong, clinically relevant potential, MPM use in HD research and clinical practice remains underexplored. Therefore, we used MPM to assess microstructural differences in the cortex, white matter (WM), and BG among manifesting PwHD (PwHD_man_), premanifest PwHD (PwHD_pre_), and healthy controls (HC). Given evidence of widespread cortical neurodegeneration in HD, we hypothesized that MPM insights would reveal cortical and BG atrophy and demyelination associated with clinical measures of HD severity [11, 12].

## Methods

### Experimental design and participants

We recruited PwHD from outpatient clinics at the Center for Rare Diseases and the inpatient clinics of the Department of Neurology, University Medical Center Schleswig–Holstein (Campus Lübeck). We selected PwHD based on a genetically confirmed diagnosis with fully penetrant, ≥ 40 CAG repeats in the HTT gene [13]. We classified participants with genetically confirmed HD as premanifest in the absence of unequivocal motor signs and as manifest when unequivocal HD-related motor signs were present. By contrast, we recruited healthy controls from pre-existing research cohorts at our institution. We determined eligibility as healthy controls from medical history and screening information collected at cohort enrollment; we included individuals only if they had no history of genetic or idiopathic neurological disease, alcohol or substance abuse, structural brain disease, or MRI contraindications. All PwHD underwent clinical examinations, including the Unified Huntington’s Disease Rating Scale, Total Motor Score (UHDRS-TMS), and cognitive screening with the Montreal Cognitive Assessment (MoCA) [14, 15]. MoCA was not administered to healthy controls. Additionally, we report Total Functional Capacity (TFC) and CAG-Age Product (CAP) scores. CAP scores were calculated using the previously reported formula [16],

CAP = Age × (CAG repeat length – 33.66)

The study adhered to the Declaration of Helsinki and was approved by the Committee for the Ethics of Safety- and Security-Relevant Research at the University of Luebeck (AZ-18–196). All participants provided informed written consent prior to study participation.

Because PwHD_pre_ and PwHD_man_ differed in mean age and sex distribution, we stratified the corresponding HC groups accordingly, ensuring each HC group matched its comparison group in age, sex distribution, and sample size. We derived the HC_pre_ group, which we compared with PwHD_pre,_ and the HC_man_ group, which we compared with PwHD_man._ HC groups comprise only healthy participants; the “pre” and “man” labels refer only to the matched PwHD group.

### Image acquisition, voxel-based quantification, and voxel-based morphometry

MRI sessions were conducted using a 3 T Siemens Magnetom Skyra scanner equipped with a 64-channel head coil. Using a multi-echo 3D fast low-angle shot, we acquired T1, PD, and magnetization transfer (MT) weightings [17]. We measured all sequences in coronal orientation with 240 contiguous slices, 1.0 mm isotropic resolution, a field of view of 256 × 176 mm^2^ (phase FoV 68.8%), GRAPPA acceleration factor 2 with 24 reference lines, and partial Fourier 6/8 in the slice direction. The received bandwidth for all echoes was 440 Hz/px. We enabled inline distortion correction (2D mode) and prescan normalization. The T1-weighted acquisition (TR = 19.0 ms, flip angle = 20°, six echoes with TE = 2.20/4.70/7.20/9.70/12.20/15.00 ms) required about 8 min. The PD-weighted acquisition (TR = 24.0 ms, flip angle = 6°, eight echoes with TE = 2.20/4.70/7.20/9.70/12.20/15.00/17.70/20.00 ms) required approximately 7 min. The magnetic transfer-weighted acquisition (TR = 37.0 ms, flip angle = 6°, six echoes with TE = 2.20/4.70/7.20/9.70/12.20/15.00 ms, with an off-resonance magnetization transfer pre-pulse enabled) required about 11 min. A neuroradiologist reviewed the neuroimaging data to exclude concurrent conditions. We visually assessed artifacts (e.g., head motion) and performed quantitative evaluations, including counting artifact voxels (exclusion threshold: artifacts exceeding two standard deviations above the mean).

Voxel-based quantification (VBQ) analysis was performed in the hMRI toolbox within the Statistical Parametric Mapping 25 framework (SPM25, Wellcome Trust Center for Neuroimaging, London) in MATLAB software (R2025a, The MathWorks Inc., Natick, MA, USA) [8]. We reconfigured the toolbox with a custom-written, scanner-specific settings script in MATLAB. We created and processed R1, R2*, PD, and MTsat maps from unprocessed, multi-echo T1-, PD-, and MT-weighted images, using the default hMRI pipeline with ‘UNICORT’ setting for B_1_ correction and unified segmentation for radiofrequency sensitivity bias correction [8]. We used the hMRI toolbox pipeline for processing, comprising segmentation (into gray matter (GM) and WM); nonlinear spatial registration to MNI space; and tissue-weighted smoothing [18]. We set a voxel size of 1 × 1 × 1 × mm^3^ and Gaussian smoothing with a 6 × 6 × 6 mm FWHM kernel.

For voxel-based morphometry (VBM) analysis, we utilized the Computational Anatomy Toolbox 12 (CAT12.9) and R1 maps generated during VBQ analysis [19].

### Statistical analyses

Using SPM25, we conducted VBQ and VBM second-level analyses involving a two-sample t-test. Age and sex were included as covariates for VBQ, and total intracranial volume was additionally included for VBM analyses. The cluster-forming threshold was set to p < 0.001 (uncorrected), with a minimum number of voxels of 20; additional cluster-level family-wise error (FWE) correction of p < 0.05 was applied. We tested two contrasts: clusters with higher values in PwHD (PwHD > HC) and clusters with lower values in PwHD (PwHD < HC). Using MarSBaR (v0.45), we extracted clusters with significant group-level differences and visualized them using Python (v3.13.5, Python Software Foundation) using libraries such as Nilearn (v.0.12.0) [20, 21]. We performed two sets of group comparisons. The two-group comparison comprised all PwHD participants (PwHDtotal; PwHD_pre_ and PwHD_man_) versus age- and sex-matched HC_total_. The four-group comparison comprised PwHD_pre_ versus HC_pre_; and PwHD_man_ versus HC_man_.

We performed separate analyses for the whole brain and the BG; VBQ and VBM; each MPM map; and GM and WM, using explicit masks. We used tissue probability masks incorporated within the SPM25 environment, and the BG mask was created using the ATAG atlas [22]. Significant clusters were anatomically labeled using the Neuromorphometrics atlas for GM and the JHU White-Matter Tractography Atlas for WM [23]. Clusters within each modality and atlas group were corrected for multiple comparisons using family-wise error (FWE) corrections. Clusters surviving the FWE-corrected threshold of pFWE < 0.05 were considered significant and are reported in this work.

Correlation analyses were performed using Pearson’s or Spearman’s correlation tests, depending on the distribution of the data. Pearson’s correlation coefficient (r) was used when the analyzed variables were approximately normally distributed and their relationship was approximately linear. Spearman’s rank correlation coefficient (rho) was used when these assumptions were not met. For every correlation, we report the analyzed group, sample size, the coefficient, and the corrected p-value. Multiple-comparison correction was performed using the Benjamini–Hochberg false discovery rate (FDR) procedure across all tests of cross-modal overlap (n = 21) and associations with clinical measures (n = 115). Disease duration was defined as the time elapsed since clinical motor onset and was analyzed only in PwHDman (n = 10). PwHDpre were excluded from analyses involving disease duration because clinical motor onset had not yet occurred.

## Results

### Demographic and Clinical Assessment

We included 26 PwHD_total_ (age: 47.1 ± 14.9 years; 15 females) and 42 HC_total_ (age: 49.0 ± 18.0 years; 22 females). Mean age (t = 0.467, df = 60, p = 0.3173) and sex distribution (chi-squared = 0.056, df = 1, p = 0.8130) did not differ between groups. Within PwHD_total_, 16 participants were PwHD_pre_ (age: 39.3 ± 12.3 years; 9 females) and 10 were PwHD_man_ (age: 59.8 ± 8.5 years; 6 females), with no age or sex differences relative to their corresponding HC groups (PwHD_pre_: age, t = 0.227, df = 31, p = 0.9820; sex distribution, chi-square = 0.022, df = 1, p = 0.8812; PwHD_man_: age, t = 0.314, df = 17, p = 0.7573; sex distribution, chi-square = 0.000, df = 1, p = 1.000). The average CAG repeat length was 42 ± 2 in PwHD_pre_ and 41 ± 1 in PwHD_man_. Mean disease duration and UHDRS-TMS scores in PwHD_man_ were 7.7 ± 5.5 years and 42.40 ± 19.72, respectively. Mean TFC scores were 12.7 ± 0.6 in PwHD_pre_ and 8.0 ± 2.5 in PwHD_man_. CAP scores were 321.6 ± 78.7 in PwHD_pre_ and 461.1 ± 66.0 in PwHD_man_. Average MoCA scores were 26.56 ± 2.59 in PwHD_pre_ and 19.70 ± 5.38 in PwHD_man_ (Table 1). UHDRS-TMS, TFC, and CAP were not done in healthy controls. MoCA was not done in healthy controls and is therefore reported as unavailable.

**Table 1:** Demographic and clinical characteristics

Two-group comparison			
	PwHD_total_	HC_total_	p-value
number	26	42	
male/female	11/15	20/22	0.8130 ^1^
age (years)	47.1 ± 14.9	49.0 ± 18.0	0.3173 ^2^
m*HTT* (no. of CAG repeats)	42 ± 2	n.a	
UHDRS-TMS	17.50 ± 23.35	n.a	
TFC	10.9 ± 2.8	n.a	
CAP	375.2 ± 100.4	n.a	
Disease duration (years)	7.7 ± 5.5 (n = 10)	n.a	
MoCA	23.92 ± 5.10	n.av	

Four-group comparison						
	PwHD_pre_	HC_pre_	p-value	PwHD_man_	HC_man_	p-value
number	16	16		10	10	
male/female	7/9	7/9	0.8812^1^	4/6	4/6	1.0000 ^1^
age (years)	39.2 ± 12.3	39.3 ± 14.5	0.9820 ^2^	59.8 ± 8.5	60.9 ± 7.1	0.7573 ^2^
m*HTT* (no. of CAG repeats)	42 ± 2	n.a		41 ± 1	n.a	
UHDRS-TMS	1.94 ± 1.98	n.a		42.40 ± 19.72	n.a	
TFC	12.7 ± 0.6	n.a		8.0 ± 2.5	n.a	
CAP	321.6 ± 78.7	n.a		461.1 ± 66.0	n.a	
Disease duration (years)	n.a	n.a		7.7 ± 5.5	n.a	
MoCA	26.56 ± 2.59	n.av		19.70 ± 5.38	n.av	

Values are mean ± standard deviation. Superscripts indicate: 1 Pearson chi-square test; 2 two-sample t-test. HCpre and HCman comprise exclusively healthy participants; “pre” and “man” identify the PwHD group to which the controls were matched. Disease duration is not defined for PwHDpre; the PwHDtotal disease-duration value therefore reflects PwHDman only (n = 10). MoCA was not done in healthy controls and is reported as unavailable. CAP: CAG-Age Product, HC: *HC* healthy controls, mHTT: number of CAG repeats in the expanded huntingtin gene, *MoCA:* Montreal Cognitive Assessment. n.a.: not applicable. n.av.: not available, *PwHDman:* manifesting participants with Huntington’s disease, *PwHDpre:* premanifest participants with Huntington’s disease, *PwHDtotal:* all premanifest and manifesting participants with Huntington’s disease, *TFC:* Total Functional Capacity, *UHDRS-TMS:* Unified Huntington’s Disease Rating Scale Total Motor Score

### Widespread microstructural cortical and subcortical reductions in patients with Huntington’s disease

**Fig. 1:**
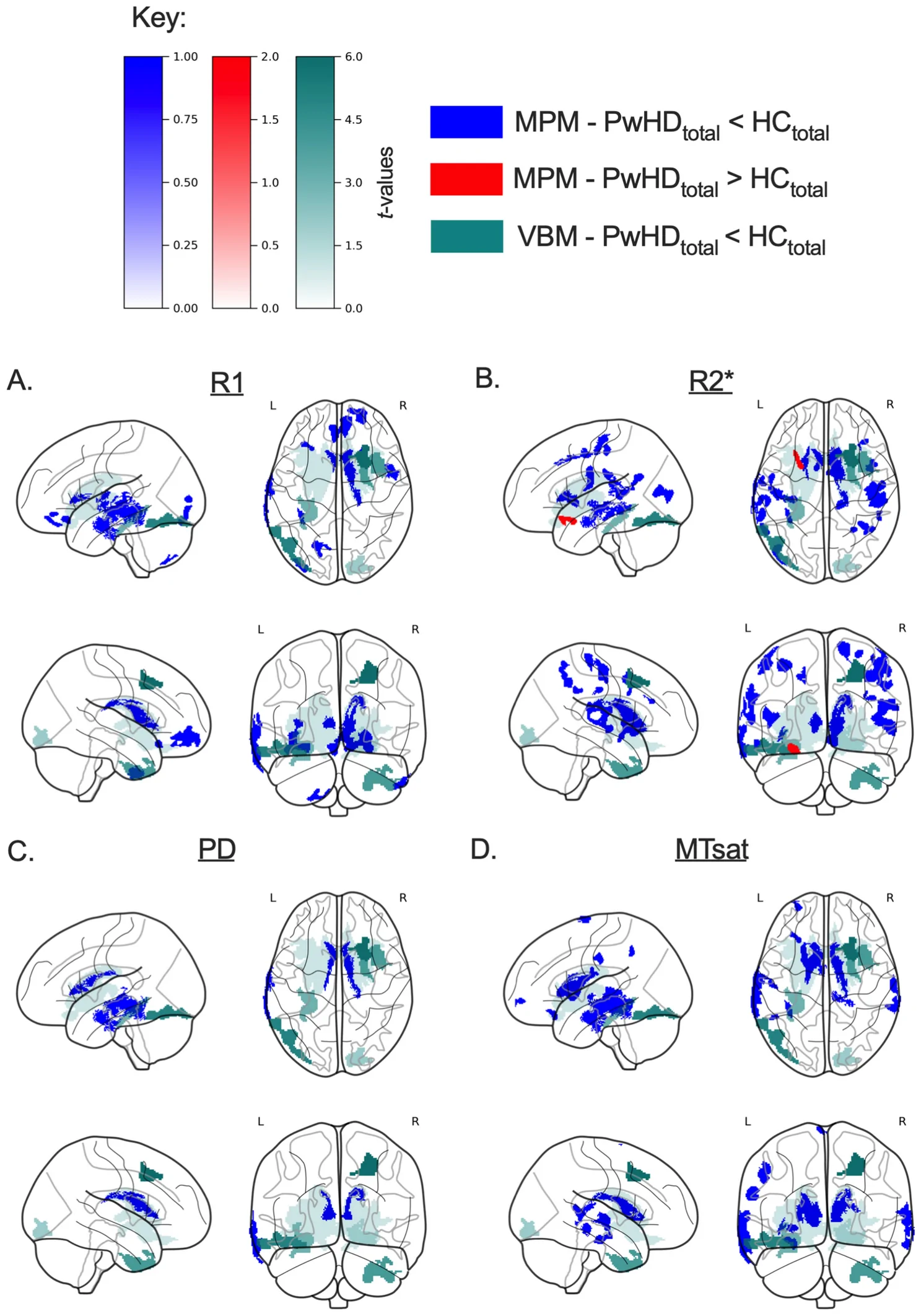
Group-level differences across MPM modalities in gray matter between PwHD_total_ and HC_total_. Voxel-wise statistical parametric maps from group comparison of MPM modalities are presented on a standard brain template in sagittal, axial, and coronal views. Color bars present voxel-wise *t*-values, with higher absolute values indicating stronger group differences. The maps are overlaid with VBM-derived clusters, presenting lower *t-*values in PwHD_total_ (in teal, T = 3.23, FWEc = 652, df = [1, 62]). A. Clusters with lower R1 values in PwHD_total_ (in blue, T = 3.22, FWEc = 157, df = [1, 64]). B. Clusters with lower R2* values (in blue) and clusters with higher R2* values in PwHD_total_ (in red, T = 3.22, FWEc = 151, df = [1, 64]). C. Clusters with lower PD values in PwHD_total_ (in blue, T = 3.22, FWEc = 402, df = [1, 64]). D. Clusters with lower MTsat values in PwHD_total_ (in blue, T = 3.22, FWEc = 170, df = [1,64]). df: degrees of freedom. FWE: family-wise error. HC_total_: healthy controls. L: left hemisphere. MPM: multiparametric mapping. MTsat: magnetization transfer saturation. PD: proton density. PwHD_total_: premanifest and manifesting patients with Huntington’s disease. R: right hemisphere. R1: longitudinal relaxation rate. R2*: effective transverse relaxation rate. T: *t*-statistic. VBM: volume-based morphometry

We observed constant reductions in *t*-values across all four MPM modalities in PwHD_total_ (Fig. 1, Tables S1-4). Identified clusters included bilateral middle temporal gyri (left: R1: T = 5.43, p_FWE_ = 0.000; R2*: T = 3.93, p_FWE_ = 0.005; PD: T = 5.64, p_FWE_ = 0.000. Right: R1: T = 5.33, p_FWE_ = 0.001; MTsat: T = 4.83, p_FWE_ = 0.000), superior temporal gyri (left: R2*: T = 4.99; MTsat: T = 6.00; right: R2*: T = 4.82; MTsat: T = 4.52; all p_FWE_ = 0.000), left posterior orbital gyrus (R1: T = 3.87, p_FWE_ = 0.000; R2*: T = 4.16, p_FWE_ = 0.000: MTsat: T = 4.38, p_FWE_ = 0.002), left fusiform gyrus (R1: T = 6.68, p_FWE_ = 0.041; R2*: T = 6.62, p_FWE_ = 0.010; MTsat: T = 6.48, p_FWE_ = 0.023), left supramarginal gyrus (R2*: T = 5.19, p_FWE_ = 0.000; MTsat: T = 4.76, p_FWE_ = 0.021) and left postcentral gyrus (R1: T = 4.14, p_FWE_ = 0.000, R2*: T= 5.78, p_FWE_ = 0.000; MTsat: T = 4.28, p_FWE_ = 0.001). Additionally, reductions in PD were observed in bilateral caudate nuclei (left: T = 4.77; right: T = 4.50; all p_FWE_ = 0.000). VBM analysis revealed only clusters with lower *t*-values in PwHD_total,_ including left caudate nucleus (T = 5.60, p_FWE_ = 0.000) and right middle frontal gyrus (T = 4.99, p_FWE_ = 0.024), which overlapped with VBQ-identified clusters (Table S5).

Within BG, *t-*values were lower in PwHD_total_ across all MPM modalities in the bilateral caudate nuclei (left: R1: T = 3.63, p_FWE_ = 0.041; R2*: T = 3.86, p_FWE_ = 0.017; MTsat: T = 4.33, p_FWE_ = 0.000. Right: R1: T = 4.07, p_FWE_ = 0.000; R2*: T = 4.40, p_FWE_ = 0.000; PD: T = 3.86, p_FWE_ = 0.040; MTsat: T = 4.14, p_FWE_ = 0.000, Figures S1-2 and Table S6). VBM-derived clusters with lower *t-*values were identified in the bilateral caudate nuclei (left: T = 5.27; right: T = 4.59; all p_FWE_ = 0.000).

### Altered R2*, R1, and MTsat in premanifest patients with Huntington’s disease

**Fig. 2:**
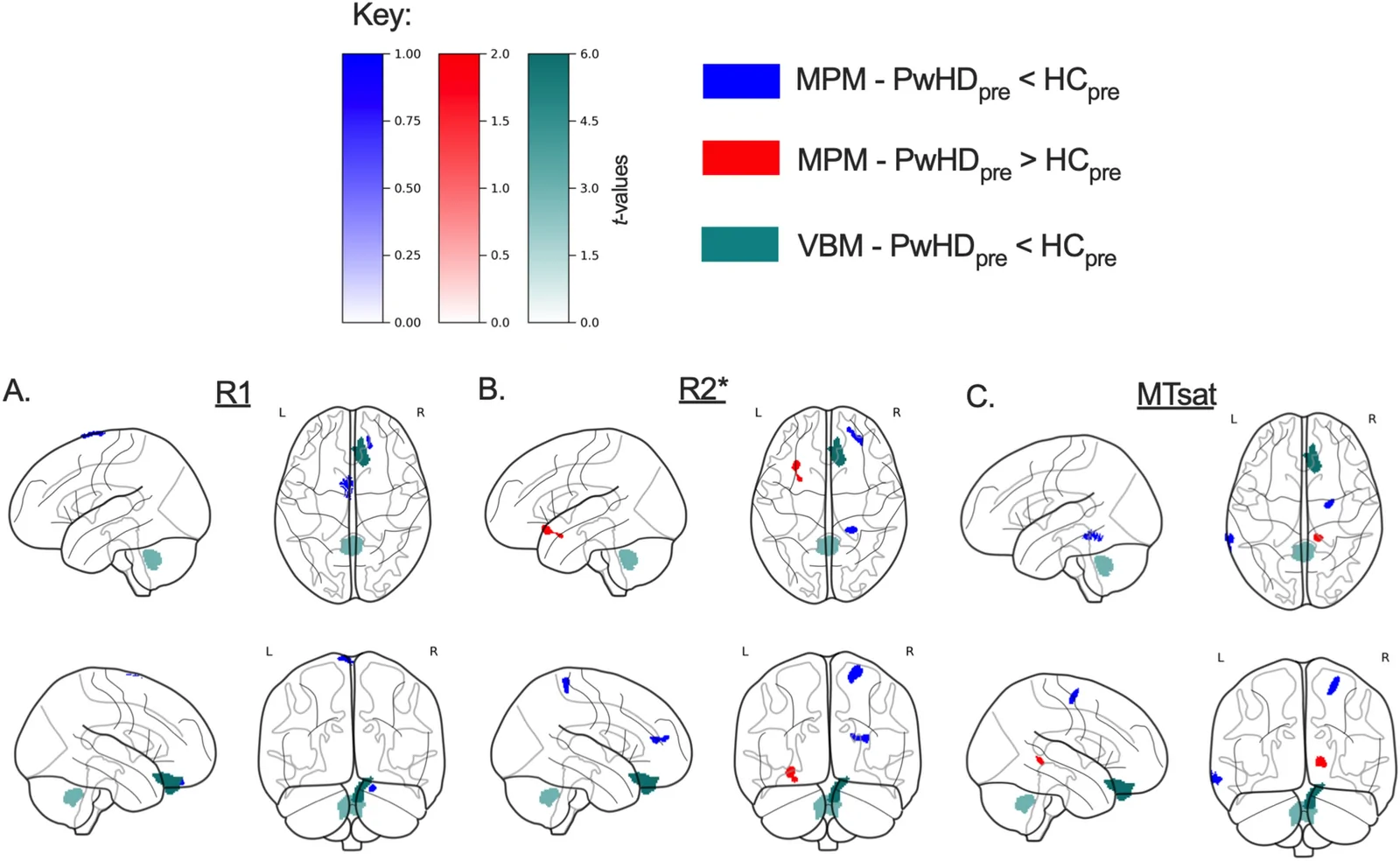
Group-level differences across MPM modalities in gray matter between PwHDpre and HCpre. Voxel-wise statistical parametric maps from group comparison of MPM modalities are presented on a standard brain template in sagittal, axial, and coronal views. Color bars present voxel-wise *t*-values, with higher absolute values indicating stronger group differences. The maps are overlaid with VBM-derived clusters, presenting lower *t-*values in PwHD_pre_ (in teal; T = 13.61, FWEc = 559, df = [1, 27]). A. Clusters with lower R1 values in PwHD_pre_ (in blue; T = 3.40, FWEc = 188, df = [1, 29]). B. Clusters with lower R2* values (in blue) and clusters with higher R2* values in PwHD_pre_ (in red; T = 3.40, FWEc = 324, df = [1, 29). C. Clusters with lower MTsat values (in blue) and clusters with higher MTsat values in PwHD_pre_ (in red, T = 3.40, FWEc = 212, df = [1, 29]). The HC_pre_ group comprises only healthy participants, and “pre” labeling refers to the matched PwHD_pre_ premanifest patients with Huntington’s disease. df: degrees of freedom, FWE: family-wise error, HC_pre_: healthy controls, L: left hemisphere, MPM: multiparametric mapping, MTsat: magnetization transfer saturation, PwHD_pre_: premanifest patients with Huntington’s disease, R: right hemisphere, R1: longitudinal relaxation rate, R2*: effective transverse relaxation rate, T: *t*-statistic, VBM: voxel-based morphometry

PwHD_pre_ presented with differences in R1, R2*, and MTsat (Fig. 2; Table S7), including lower R1 values in the left superior frontal gyrus (T = 5.53, p_FWE_ = 0.028) and left middle temporal gyrus (T = 3.95, p_FWE_ = 0.031); lower R2* values in the right middle frontal gyrus (T = 5.26, p_FWE_ < 0.001) and right superior parietal lobule (T = 4.84, p_FWE_ < 0.001); and lower MTsat values in the right superior frontal gyrus (T = 5.82, p_FWE_ = 0.001) and right posterior cingulate gyrus (T = 4.45, p_FWE_ = 0.014). VBM analysis showed that PwHD_pre_ had lower t-values in cerebellar vermal lobules (T = 28.76, p_FWE_ < 0.001) and the right middle orbital gyrus (T = 23.26, p_FWE_ = 0.007) than HC_pre_. No significant group differences were observed in basal-ganglia VBQ or VBM analyses.

### Multimodal reductions in cortical and subcortical structures in manifesting patients with Huntington’s disease

**Fig. 3:**
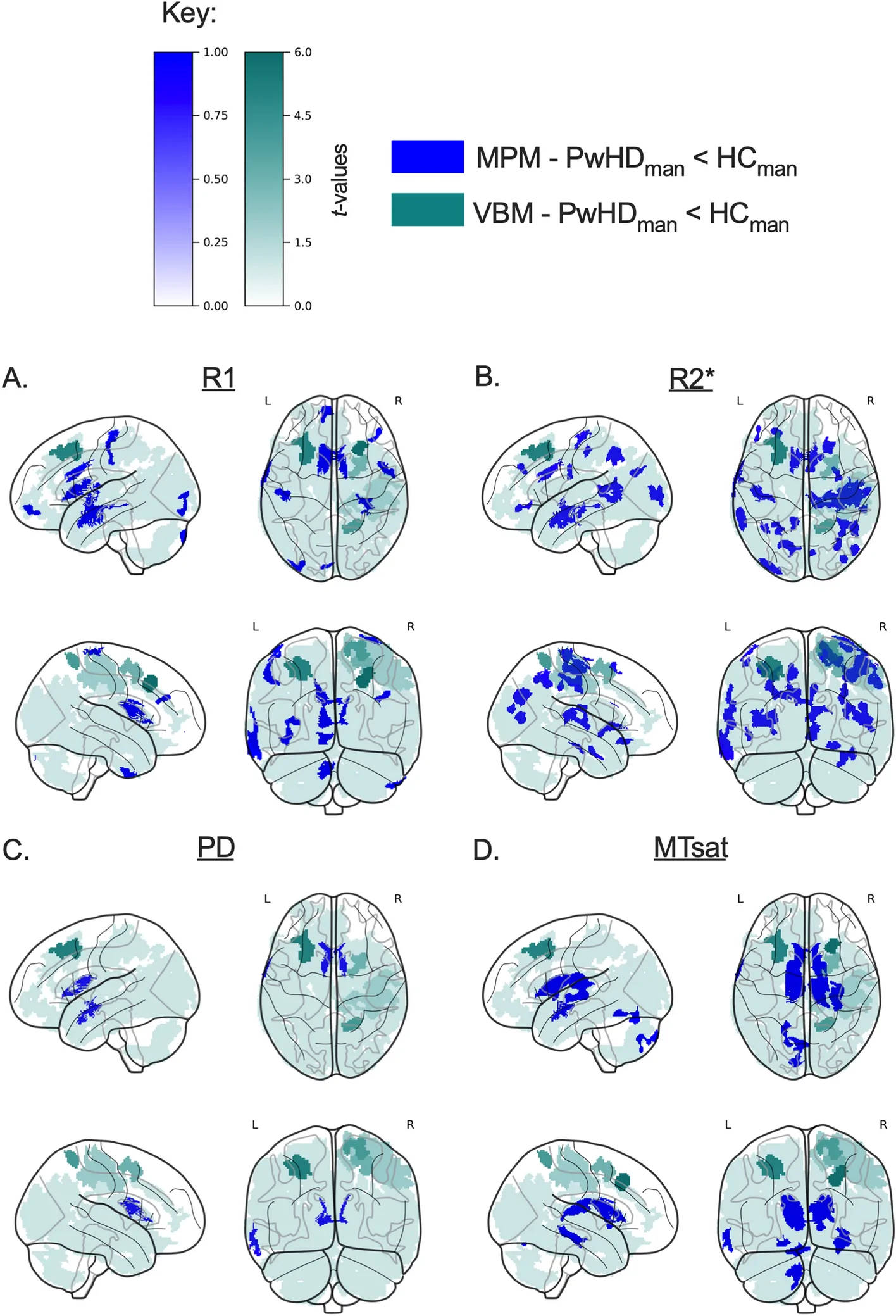
Group-level differences across MPM modalities in gray matter between PwHD_man_ and HC_man_. Voxel-wise statistical parametric maps from group comparison of MPM modalities are presented on a standard brain template in sagittal, axial, and coronal views. Color bars present voxel-wise *t*-values, with higher absolute values indicating stronger group differences. The maps are overlaid with VBM-derived clusters, presenting lower *t*-values in PwHD_man_ (in teal, T = 3.79, FWEc = 445, df = [1, 14]. A. Clusters with lower R1 values in PwHD_man_ (in blue, T = 3.69, FWEc = 203, df = [1, 16]). B. Clusters with lower R2* values in PwHD_man_ (in blue, T = 3.69, FWEc = 191, df = [1, 16]). C. Clusters with lower PD values in PwHD_man_ (in blue, T = 3.69, FWEc = 275, df = [1, 16]). D. Clusters with lower MTsat values in PwHD_man_ (in blue, T = 3.69, FWEc = 282, df [1, 16]). The HC_man_ group comprises only healthy participants, and “man” labeling refers to the matched PwHD_man_ group. df: degrees of freedom, FWE: family-wise error, HC_man_: healthy controls, L: left hemisphere, MPM: multiparametric mapping, MTsat: magnetization transfer saturation, PD: proton density, PwHD_man_: manifesting patients with Huntington’s disease, R: right hemisphere, R1: longitudinal relaxation rate, R2*: effective transverse relaxation rate, T: *t-*statistic, VBM: volume-based morphometry

All identified clusters presented with lower *t-*values in PwHD_man_ (Fig. 3; Tables S8-11). The left superior temporal gyrus showed a consistent reduction across all MPM modalities (R1: T = 10.65; R2*: T = 5.09; all p_FWE_ = 0.000; PD: T = 6.96; MTsat: T = 10.72; all p_FWE_ = 0.016). Multiple clusters demonstrated reduced R1 and R2* values, including the bilateral postcentral gyrus (left: R1: T = 8.21, p_FWE_ = 0.000; R2*: T = 6.67, p_FWE_ = 0.002. Right: R1: T = 6.71, p_FWE_ = 0.028; R2*: T = 6.60, p_FWE_ = 0.000), the left middle cingulate gyrus (R1 = 8.20, p_FWE_ = 0.003; R2*: T = 8.10, p_FWE_ = 0.002), and the left inferior occipital gyrus (R1 = 4.77; R2*: T = 9.14, all p_FWE_ = 0.000). Lower MTsat (all p_FWE_ = 0.000) values were detected in cerebellar structures, including left cerebellum exterior (T = 4.65) and vermal lobules VII-VIII (T = 7.16). All VBM-identified clusters overlapped with VBQ-derived clusters, including within the frontal (right superior frontal gyrus: T = 7.33, p_FWE_ = 0.001; right middle frontal gyrus: T = 6.45, p_FWE_ = 0.023; and left middle frontal gyrus: T = 6.42, p_FWE_ = 0.000) and parietal (right superior parietal lobule: T = 7.33, p_FWE_ = 0.023; and right postcentral gyrus: T = 10.46, p_FWE_ = 0.000) lobes (Table S12). Furthermore, the largest VBM-identified clusters in the right fusiform gyrus (T = 16.32, p_FWE_ = 0.000) overlapped with lower R2* values (T = 5.51, p_FWE_ = 0.033).

**Fig. 4:**
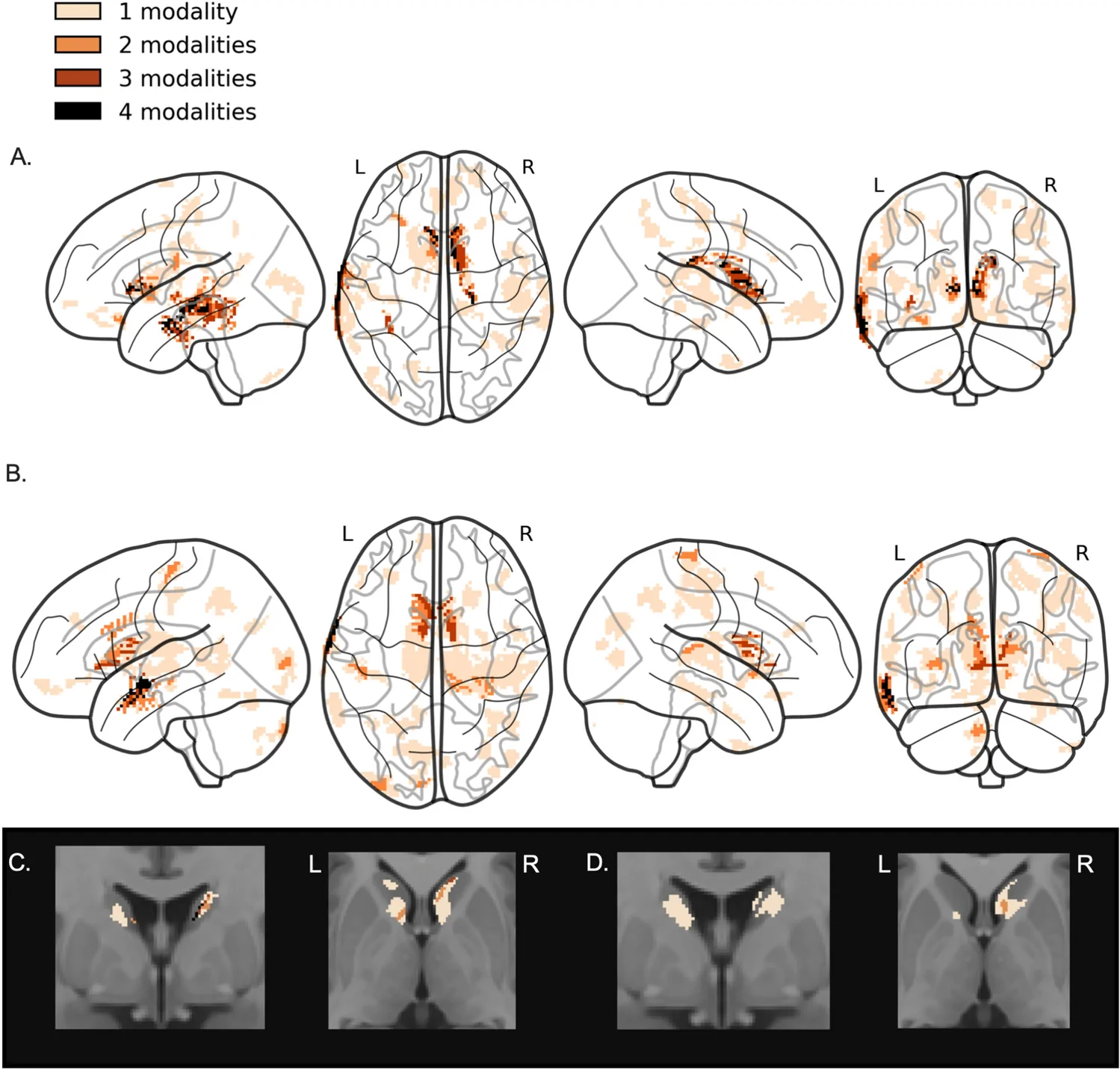
Venn-encoded whole-brain gray matter and basal ganglia overlap of MPM-derived statistical parametric maps. The figure presents overlaid voxel-wise maps of group differences across MPM modalities, color-coded by the number of cross-modal overlaps. A. Overlap of R1, R2*, PD, and MTsat group differences between PwHD_total_ and HC_total_. B. Overlap of R1, R2, PD, and MTsat differences in PwHD_man_ and HC_man._ C. Overlap of R1, R2*, PD, and MTsat group differences in the basal ganglia between PwHD_total_ and HC_total._ D. Overlap of R2* and MTsat group differences in the basal ganglia between PwHD_man_ and HC_man_. The HC_man_ group comprises only healthy participants, and “man” labeling refers to the matched PwHD_man_ group. HC_total/man_: healthy controls, L: left hemisphere, MPM: multiparametric mapping, MTsat: magnetization transfer saturation, PD: proton density, PwHD_man_: manifesting patients with Huntington’s disease, PwHD_total_: premanifest and manifesting patients with Huntington’s disease, R: right hemisphere, R1: longitudinal relaxation rate, R2*: effective transverse relaxation rate

The BG analysis showed reduced R2* and MTsat values only in PwHD_man_ (Figure S3 and Table S13). The right caudate nucleus showed lower R2* (T = 4.75, p_FWE_ = 0.001) and MTsat values (T = 5.23, p_FWE_ = 0.000), while the left caudate nucleus showed reduced MTsat values only (T = 6.06, p_FWE_ = 0.000). All VBQ-derived clusters overlapped with VBM-derived reductions (all p_FWE_ = 0.000) in the right (T = 5.37) and left putamen (T = 5.07) (Fig. 4).

### Overlapping and correlated cross-modal group differences within the same clusters

In PwHD_total_ (n = 26), reductions across MPM modalities overlapped within the left fusiform gyrus, left middle temporal gyrus, right thalamus, and left middle occipital gyrus (Table S14). Cross-modal correlations used subject-level cluster means; the correction status of all p-values in this paragraph is FDR-corrected. Within clusters showing group-level reductions, R1 values correlated positively with R2* values in the left fusiform gyrus (rho = 0.953, p_FDR_ = 0.0002), right thalamus (rho = 0.955, p_FDR_ = 0.0002), left caudate nucleus (r = 0.690, p_FDR_ = 0.0002), right caudate nucleus (r = 0.694, p_FDR_ = 0.0002), and left middle occipital gyrus (r = 0.551, p_FDR_ = 0.0043). R1 values also correlated positively with MTsat values in the left fusiform gyrus (r = 0.836, p_FDR_ = 0.0002), right thalamus (r = 0.707, p_FDR_ = 0.0002), left caudate nucleus (rho = 0.910, p_FDR_ = 0.0002), and right caudate nucleus (rho = 0.934, p_FDR_ = 0.0002). R1 and PD values correlated positively in the left middle temporal gyrus (rho = 0.966, p_FDR_ = 0.0002). R2* and MTsat values correlated positively in the left fusiform gyrus (rho = 0.892, p_FDR_ = 0.0002), right thalamus (rho = 0.691, p_FDR_ = 0.0002), left caudate nucleus (rho = 0.753, p_FDR_ = 0.0002), and right caudate nucleus (rho = 0.837, p_FDR_ = 0.0002).

In PwHD_man_ (n = 10), R1 and R2* values correlated positively in the left superior temporal gyrus (rho = 0.660, p_FDR_ = 0.0438), left postcentral gyrus (rho = 0.878, p_FDR_ = 0.0021), left middle cingulate gyrus (rho = 0.987, p_FDR_ = 0.0002), right postcentral gyrus (rho = 0.927, p_FDR_ = 0.0004), and left inferior occipital gyrus (rho = 0.745, p_FDR_ = 0.0203). In the left superior temporal gyrus, R1 and MTsat values also correlated positively (rho = 0.672, p_FDR_ = 0.0410).

### Associations between MPM-derived values and clinical variables

Clinical correlations were calculated at the subject level in PwHD_total_ (n = 26); the correction status of all p-values in this paragraph is FDR-corrected (Table S15). R2* values within clusters characterized by group-level reductions correlated positively with MoCA scores in the right middle occipital gyrus (rho = 0.927, p_FDR_ = 0.0005) and left precuneus (rho = 0.884, p_FDR_ = 0.0144). Thus, higher R2* values were associated with higher MoCA scores. CAG repeat length correlated positively with R1 and R2* values in the left middle cingulate gyrus (R1: rho = 0.636, p_FDR_ = 0.0492; R2*: rho = 0.636, p_FDR_ = 0.0492), left supramarginal gyrus (rho = 0.532, p_FDR_ = 0.0111), and right precentral gyrus (rho = 0.395, p_FDR_ = 0.0103), and MTsat values in the left superior temporal gyrus (rho = 0.399, p_FDR_ = 0.0473).

### VBQ- and VBM-derived white matter microstructural and volumetric changes

We observed multimodal MPM reductions in PwHD_total_ and PwHD_pre_. No significant white-matter VBM differences were detected in either group (Figures S4-7 and Tables S16-19). Within the PwHD_man_ group, we detected MPM reductions (all p_FWE_ = 0.000), within the splenium (R1: T = 21.58; R2*: T = 57.4; MTsat: T = 5.09) and genu of the corpus callosum (R1: T = 6.56; R2*: T = 6.16; MTsat: T = 5.83) (Figure S4 and Table S16). Consistent with GM findings, MPM-derived clusters overlapped with VBM-derived reductions in *t-*values in PwHD_man_ in posterior corona radiata (left: T = 7.52, p_FWE_ = 0.011; right: T = 5.65, p_FWE_ = 0.015), right superior corona radiata (T = 6.16, p_FWE_ = 0.016), sagittal stratum (T = 6.26, p_FWE_ = 0.000), and left cingulum (T = 6.11, p_FWE_ = 0.000). Some of the clusters with identified VBQ reductions cross-modally overlapped (Figure S7).

## Discussion

Utilizing MPM, we detected microstructural alterations in PwHD. PwHD_total_ showed widespread cortical and subcortical reductions across all MPM parameters, whereas PwHD_man_ showed more spatially constrained changes with stronger group effects. Reductions in R1 and MTsat are consistent with lower myelin and macromolecular content, and reduced R2* may reflect lower iron content and/or demyelination. Proton density provides a semiquantitative measure of the MR-visible mobile proton pool and is influenced by both tissue water and macromolecular composition. The observed reductions in PD should therefore not be interpreted as direct evidence of reduced tissue water content. Although changes in tissue water may contribute to PD alterations, their direction cannot be inferred from PD measurements alone. The cross-modal overlap of MPM-derived findings was spatially consistent with VBM-derived cortical and subcortical gray- and white-matter volume loss.

Bilateral caudate nuclei consistently demonstrated reductions across all MPM maps in PwHD_man_, while reductions in R2* and MTsat in PwHD_pre_ suggest sensitivity to pathology before motor manifestation. Although R2* was reduced at the group level in the identified regions, the positive within-group correlations indicate that participants with higher individual R2* values had higher MoCA scores. Subject-level correlations also linked regional MPM values with CAG repeat length. Whole-brain VBM in PwHD_pre_ showed significant reduction in gray matter volume in the right middle orbital gyrus. Within the basal ganglia, we observed no MPM- or VBM-derived differences in PwHD_pre_, possibly because of limited sensitivity to subtle changes or limited statistical power (n = 16) [24]. Alternatively, the basal ganglia are functionally segregated into motor, associative, and limbic circuits, and analyses using functional atlases may provide greater sensitivity [25]. Although quantitative susceptibility mapping studies have demonstrated iron accumulation in the basal ganglia in PwHD, we observed reduced R2*, which may reflect lower iron content [26]. QSM and R2* differ in their sensitivity to iron and myelin, limiting direct comparison [27, 28]. Overlapping reductions across MPM modalities and VBM are consistent with combined demyelination, altered proton density, and loss of neuronal structure, which may also reduce the cellular infrastructure available for iron accumulation.

Spatially restricted clusters with higher R2* and MTsat in PwHD_pre_ may reflect local iron accumulation and neuroinflammation, indicating early-stage alterations with regional susceptibility. MPM-derived alterations were observed in the superior and middle frontaly gyrus, and middle temporal gyrus. These findings were accompanied by VBM-derived volume loss in the right middle orbital gyrus [11, 12].

The two- and four-group comparisons revealed distinct spatial patterns in MPM-related alterations in PwHD_total_ and PwHD_man_, with PwHD_total_ showing the core disease-related microstructural alterations, whereas PwHD_man_ exhibited spatially confined clusters with larger effect sizes, reflecting disease-stage vulnerability. We observed consistent reductions across all four MPM modalities in the bilateral caudate nucleus, thalamus, middle forntal gyrus, superior temporal gyrus, postcentral gyri, and occipital gyri, aligning with available evidence and adding insight into the biophysical changes and mechanisms driving the observed neurodegeneration [29, 30]. Furthermore, our findings are supported by longitudinal and postmortem studies demonstrating progressive neurodegeneration with disease progression [31–33].

Although MPM alterations in PwHD_man_ were associated with clinical measures, no single modality could be attributed to a specific clinical measure. Given the multisystem and progressive nature of HD, the identified changes are best understood as contributors rather than sole causes of the clinical presentation [34–36].

The similar pattern of reductions in WM across MPM modalities demonstrates widespread microstructural alterations in both tissue types. Although WM degeneration has been shown to precede GM loss, we did not replicate this finding and detected no white-matter volumetric loss in PwHD_pre_ [37]. Because diffusion tensor imaging (DTI) is the most widely used technique for assessing WM integrity and relies on fundamentally different physical principles than MPM, cross-comparing findings is challenging. However, observed reduction in R2* in PwHD_pre_ and PwHD_man_ may suggest that myelin changes may occur before neuronal loss in later stages of HD.

Our study has several limitations, including the cross-sectional design. We aimed to demonstrate the utility of the MPM in HD research, and future studies should include longitudinal trajectories to assess the disease-stage-dependent progression of MPM-derived findings. Additionally, stratifying disease stage into PwHD_pre_ and PwHD_man_ cohorts reduced statistical power and our ability to detect more subtle microstructural changes, particularly in PwHD_pre._ The narrow range of CAG repeat lengths within our cohort may limit our ability to detect significant correlations between repeat length and MPM-derived metrics. Therefore, future studies should aim to include larger, more heterogeneous cohorts of PwHD. The measure of disease severity used has its own limitations, including intra- and inter-rater variability and subjective bias, while MoCA tests assess the general state of cognition with no domain-specific insights [38–40]. Further studies may use a multi-domain composite UHDRS-TMS and a more representative panel of cognitive and neuropsychological assessments, as well as integrate genetic expression profiling and post-mortem investigation, to gain greater insight into the association between MPM-derived findings and specific domains of symptomatology and underlying cellular compositions [36, 41].

In conclusion, using MPM, we identified widespread cortical and subcortical microstructural alterations across HD stages, with reductions in tissue biophysical properties consistent with neurodegeneration. These observations were spatially overlapping with volumetric reductions and were associated with clinical measures in PwHD, supporting the utility of MPM for in vivo assessment of disease-related pathology in HD. Given the high volume of insights generated in a single scan, MPM has great potential to improve diagnostics and patient stratification, as well as personalized treatment approaches, though this requires confirmation in future longitudinal studies.

## Supplementary Information

Below is the link to the electronic supplementary material.

Supplementary file1 (PDF 5153 KB)

## Data Availability

The data generated in this study is not publicly available. De-identified data may be provided by the corresponding authors upon reasonable request, subject to institutional approval.
