# Supplementary material for: In vivo assessment of brain microstructure in patients with Huntington’s disease using quantitative MRI
Source: J Neurol. 2026 Nov 7;273(11):707. doi: 10.1007/s00415-026-14244-8 (PMC13744531; doi:10.1007/s00415-026-14244-8)
Supplement: Supplementary file 1 [file 415_2026_14244_MOESM1_ESM.pdf]

Table S1. Clusters with significant R1 group-level differences in the whole-brain gray matter, $PwHD_{total}$ versus HC_{total}.

	cluster level				peak level		mm	mm	mm
	$P_{FWE\text{-}corr}$	$q_{FRD\text{-}corr}$	K_E	P_{uncorr}	T	(Z_E)			
$PwHD_{total}$ < HC_{total}									
lFuG	0.041	0.006	157	0.000	6.88	5.93	-38	-38	8
lMTG	0.000	0.000	1653	0.000	5.43	4.91	-68	-6	-24
rMTG	0.001	0.000	267	0.000	5.33	4.83	56	3	-42
lCaudate	0.012	0.002	190	0.000	4.54	4.21	-7	22	3
rAOrG	0.000	0.000	1133	0.000	4.54	4.21	22	61	-9
rThal	0.028	0.005	167	0.000	4.49	4.17	20	-22	24
rCaudate	0.000	0.000	533	0.000	4.29	4.01	2	19	1
lMOG	0.010	0.002	195	0.000	4.25	3.97	-33	-92	6
lCE	0.003	0.001	232	0.000	4.22	3.95	-22	-69	-58
lPoG	0.000	0.000	316	0.000	4.14	3.88	-52	-19	37
rCaudate	0.000	0.000	318	0.000	4.12	3.87	12	-1	26
lIOG	0.000	0.000	411	0.000	4.12	3.86	-42	-85	-17
lCE	0.000	0.000	372	0.000	4.05	3.81	-30	-89	-32
rPOrG	0.002	0.001	238	0.000	4.05	3.80	25	26	-15
lPOrG	0.000	0.000	306	0.000	3.87	3.65	-33	31	-15
rMOrG	0.000	0.000	1303	0.000	3.84	3.63	11	53	-15
rCE	0.011	0.002	192	0.000	3.71	3.52	11	-87	-38
lMFC	0.000	0.000	375	0.000	3.68	3.50	-6	47	-14

FDR_{corr}: false-discovery rate-corrected. FWE_{corr}: family-wise error-corrected. HC_{total}: healthy controls. K_E: cluster extent. lCaudate: left caudate nucleus. lCE: left cerebellum exterior. lFuG: left fusiform gyrus. lIOG: left inferior occipital gyrus. lMFC: left middle frontal cortex. lMOG: left middle occipital gyrus. lMTG: left middle temporal gyrus. lPoG: left postcentral gyrus. lPOrG: left posterior orbital gyrus. $PwHD_{total}$: premanifest and manifesting patients with Huntington's disease. R1: longitudinal relaxation rate. rAOrG: right anterior orbital gyrus. rCaudate: right caudate nucleus. rCE: right cerebellum exterior. rMOrG: right middle orbital gyrus. rMTG: right middle temporal gyrus. rPOrG: right posterior orbital gyrus. rThal: right thalamus. T: *t*-statistic. Uncorr: uncorrected. Z_E: equivalent z-score.

Table S2. Clusters with significant R2* group-level differences in the whole-brain gray matter, $PwHD_{total}$ versus HC_{total}.

	cluster level				*peak level*		mm	mm	mm
	$P_{FWE\text{-}corr}$	$q_{FRD\text{-}corr}$	K_E	P_{uncorr}	T	(Z_E)			
$PwHD_{total}$ > HC_{total}									
lPOrG	0.000	0.000	409	0.000	4.16	3.90	-25	9	-18
$PwHD_{total}$ < HC_{total}									
lFuG	0.010	0.001	190	0.000	6.62	5.76	-38	-39	-8
lPoG	0.000	0.000	531	0.000	5.78	5.17	-50	-18	60
rPrG	0.000	0.000	921	0.000	5.41	4.89	48	-15	54
lSFG	0.005	0.001	209	0.000	5.35	4.85	-20	14	43
rMFG	0.033	0.003	158	0.000	5.21	4.74	22	10	42
lSMG	0.000	0.000	708	0.000	5.19	4.74	-67	-29	20
lCaudate	0.007	0.001	200	0.000	5.03	4.60	-22	21	7
lSTG	0.000	0.000	563	0.000	4.99	4.57	-70	-18	-4
rSTG	0.000	0.000	2069	0.000	4.82	4.44	54	-10	-2
rThal	0.023	0.002	167	0.000	4.79	4.41	19	-21	22
lMOG	0.000	0.000	686	0.000	4.78	4.40	-47	-73	10
lPoG	0.000	0.000	301	0.000	4.77	4.40	-62	-23	30
rAnG	0.000	0.000	834	0.000	4.77	4.39	38	-50	42
lPrG	0.000	0.000	651	0.000	4.63	4.28	-61	-9	24
rPoG	0.000	0.000	1596	0.000	4.55	4.22	37	-31	70
lCaudate	0.000	0.000	478	0.000	4.45	4.14	-4	15	4
lPrG	0.000	0.000	490	0.000	4.41	4.11	-33	-25	52
rCaudate	0.000	0.000	2689	0.000	4.39	4.09	5	16	1
rPoG	0.000	0.000	590	0.000	4.21	3.94	14	-31	68
rMFG	0.000	0.000	526	0.000	4.15	3.89	47	12	42
rMFG	0.029	0.003	161	0.000	4.14	3.88	33	30	26
rSFG	0.002	0.000	239	0.000	4.10	3.85	17	15	54
rSPL	0.043	0.004	151	0.000	4.08	3.83	28	-54	62
lPrG	0.017	0.002	176	0.000	4.08	3.83	-55	-5	47
lSTG	0.005	0.001	207	0.000	3.93	3.71	-57	-24	-14
rPoG	0.024	0.002	166	0.000	3.80	3.60	47	-20	36
rSMG	0.006	0.001	204	0.000	3.72	3.53	53	-43	36
lSMG	0.000	0.000	368	0.000	3.70	3.51	-61	-55	24

FDR_{corr}: false-discovery rate-corrected. FWE_{corr}: family-wise error-corrected. HC_{total}: healthy controls. K_E: cluster extent. lCaudate: left caudate nucleus. lFuG: left fusiform gyrus. lMOG: left middle occipital gyrus. lPoG: left postcentral gyrus. lPOrG: left posterior orbital gyrus. lPrG: left precentral gyrus. lSFG: left superior frontal gyrus. lSMG: left supramarginal gyrus. lSTG: left superior temporal gyrus. $PwHD_{total}$: premanifest and manifesting patients with Huntington's disease. $R2^*$: effective transverse relaxation rate. rAnG: right angular gyrus. rCaudate: right caudate nucleus. rMFG: right middle frontal gyrus. rPoG: right postcentral gyrus. rPrG: right precentral gyrus. rSFG: right superior frontal gyrus. rSMG: right supramarginal gyrus. rSPL: right superior parietal lobule. rSTG: right superior temporal gyrus. rThal: right thalamus. T: *t*-statistic. Uncorr: uncorrected. Z_E: equivalent z-score.

Table S3. Clusters with significant PD group-level differences in the whole-brain gray matter, $PwHD_{total}$ versus HC_{total}.

	cluster level				*peak level*		*mm*	*mm*	*mm*
	$P_{FWE\text{-}corr}$	$q_{FRD\text{-}corr}$	K_E	P_{uncorr}	T	(Z_E)			
$PwHD_{total} < HC_{total}$									
lMTG	0.000	0.000	1253	0.000	5.64	5.06	-73	-35	-9
lCaudate	0.000	0.000	402	0.000	4.77	4.39	-9	23	4
rCaudate	0.000	0.000	804	0.000	4.50	4.18	12	-1	26

FDR_{corr}: false-discovery rate-corrected. FWE_{corr}: family-wise error-corrected. HC_{total}: healthy controls. K_E: cluster extent. lCaudate: left caudate nucleus. lMTG: left middle temporal gyrus. PD: proton density. $PwHD_{total}$: premanifest and manifesting patients with Huntington's disease. rCaudate: right caudate nucleus. T: *t*-statistic. Uncorr: uncorrected. Z_E: equivalent z-score.

Table S4. Clusters with significant MTsat group-level differences in the whole-brain gray matter between $PwHD_{total}$ and HC_{total}.

	Cluster level				*peak level*		*mm*	*mm*	*mm*
	$P_{FWE\text{-}corr}$	$q_{FRD\text{-}corr}$	K_E	P_{uncorr}	*T*	(Z_E)			
$PwHD_{total} < HC_{total}$									
lFuG	0.023	0.004	189	0.000	6.48	5.66	-39	-36	-10
lSTG	0.000	0.000	3349	0.000	6.00	5.32	-70	-24	-3
lCaudate	0.000	0.000	2494	0.000	5.53	4.98	-22	20	9
lSFG	0.043	0.005	170	0.000	5.01	4.59	-3	-5	75
rMTG	0.000	0.000	514	0.000	4.83	4.44	71	-40	7
lSMG	0.021	0.004	191	0.000	4.76	4.39	-50	-49	45
rSTG	0.000	0.000	851	0.000	4.52	4.19	69	-13	1
rMTG	0.001	0.000	297	0.000	4.44	4.13	73	-29	-12
lPOrG	0.002	0.000	274	0.000	4.38	4.08	-26	28	-19
lPoG	0.001	0.000	297	0.000	4.28	4.00	-60	-9	21
rSMG	0.007	0.001	227	0.000	4.19	3.92	61	-44	23
rThal	0.000	0.000	906	0.000	4.09	3.84	19	-21	22
lPrG	0.001	0.000	315	0.000	4.07	3.82	-51	-17	-37
rHip	0.025	0.004	186	0.000	4.06	3.82	24	-38	9
lMFG	0.030	0.004	181	0.000	3.94	3.72	-28	60	-4
rCaudate	0.003	0.001	257	0.000	3.85	3.64	13	-10	24

FDR_{corr}: false-discovery rate-corrected. FWE_{corr}: family-wise error-corrected. HC_{total}: healthy controls. K_E: cluster extent. lCaudate: left caudate nucleus. lFuG: left fusiform gyrus. lMFG: left middle frontal gyrus. lPoG: left postcentral gyrus. lPOrG: left posterior orbital gyrus. lPrG: left precentral gyrus. lSFG: left superior frontal gyrus. lSMG: left supramarginal gyrus. lSTG: left superior temporal gyrus. MTsat: magnetization transfer saturation. $PwHD_{total}$: premanifest and manifesting patients with Huntington's disease. rCaudate: right caudate nucleus. rHip: right hippocampus. rMTG: right middle temporal gyrus. rSMG: right supramarginal gyrus. rSTG: right superior temporal gyrus. rThal: right thalamus. T: *t*-statistic. Uncorr: uncorrected. Z_E: equivalent z-score.

Table S5. Clusters with significant VBM group-level differences in the whole-brain gray matter, $PwHD_{total}$ versus HC_{total}.

	cluster level				peak level		mm	mm	mm
	$P_{FWE\text{-}corr}$	$q_{FRD\text{-}corr}$	K_E	P_{uncorr}	T	(Z_E)			
$PwHD_{total}$ < HC_{total}									
lCaudate	0.000	0.000	14234	0.000	5.60	5.02	-18	-16	27
rMFG	0.024	0.012	664	0.001	4.99	4.56	27	16	44
rTMP	0.002	0.002	1078	0.000	4.35	4.04	44	8	-48
rLiG	0.026	0.012	657	0.001	4.30	4.01	14	-94	-12
lITG	0.005	0.004	907	0.000	4.26	3.98	-52	-66	-18
lPHG	0.026	0.012	652	0.001	4.04	3.79	-27	-38	-15

FDR_{corr}: false-discovery rate-corrected. FWE_{corr}: family-wise error-corrected. HC_{total}: healthy controls. K_E: cluster extent. lCaudate: left caudate nucleus. lITG: left inferior temporal gyrus. lPHG: left parahippocampal gyrus. $PwHD_{total}$: premanifest and manifesting patients with Huntington's disease. rLinG: right lingual gyrus. rMFG: right middle frontal gyrus. rTMP: right temporal pole. T: *t*-statistic. Uncorr: uncorrected. VBM: volume-based morphometry. Z_E: equivalent z-score.

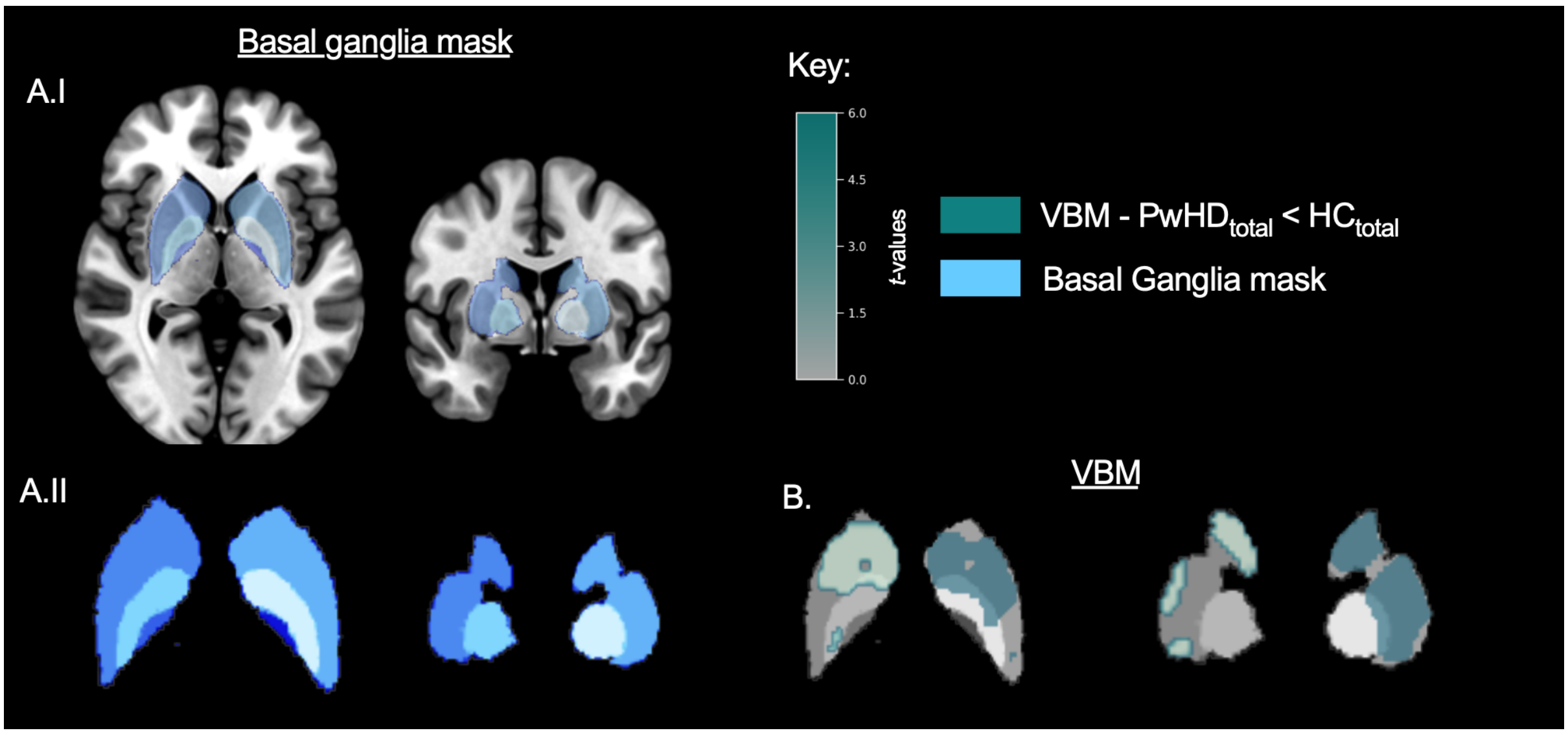

Figure S1. Group-level differences in VBM in the basal ganglia, $PwHD_{total}$ versus HC_{total}. In panel A, we present the basal ganglia mask used in MPM and VBM analysis, overlaid on the T1-weighted template (A.I) and the original mask (A.II). In panel B, the VBM results are presented, demonstrating clusters in the basal ganglia where $PwHD_{total}$ present with lower t-values (in teal, T = 3.79, FWEc = 445, df = [1, 14]. df: degrees of freedom. FWE: family-wise error. HC_{total}: healthy controls. $PwHD_{total}$: premanifest and manifesting patients with Huntington's disease. T: t-statistic. VBM: volume-based morphometry.

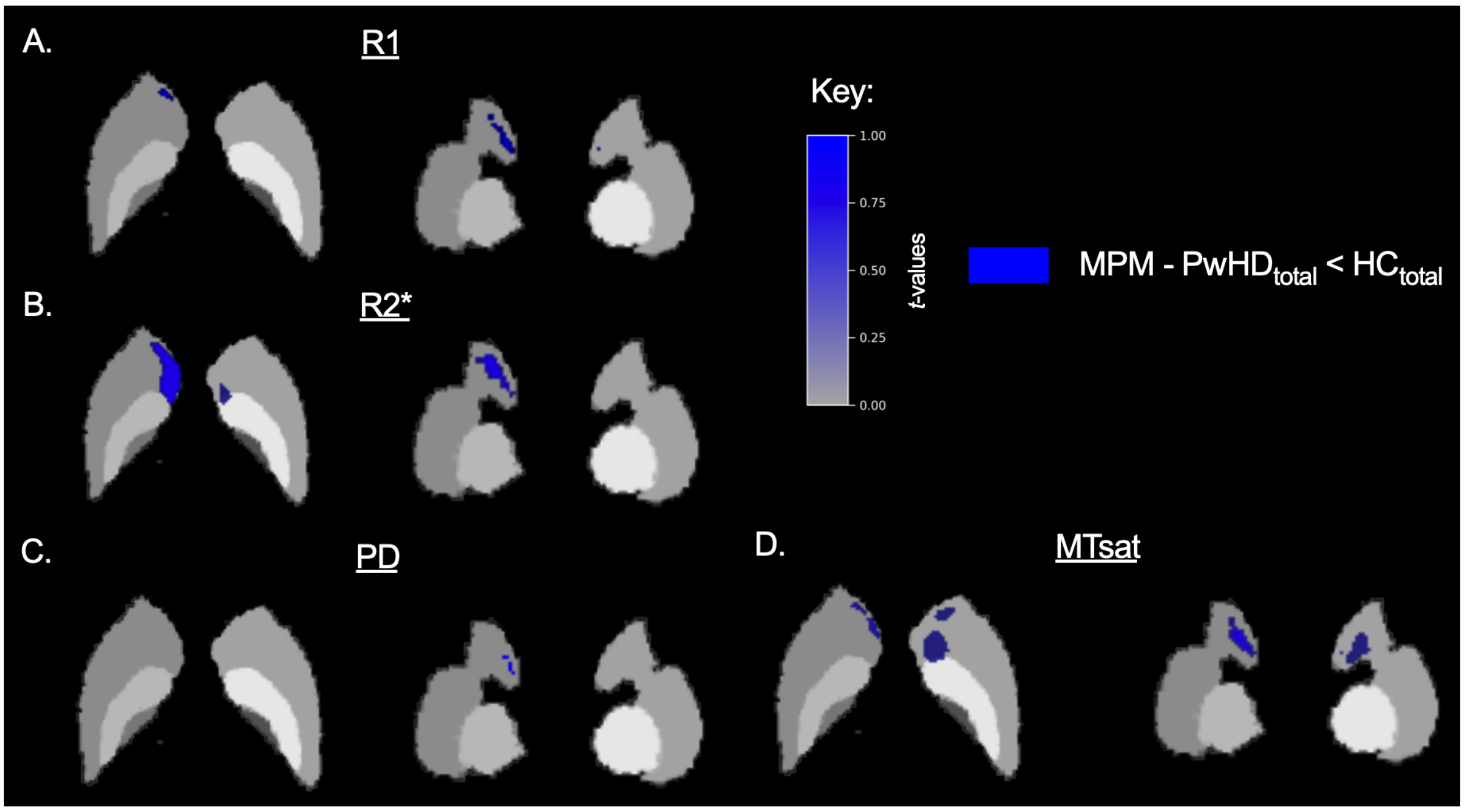

Figure S2. Group-level differences across MPM modalities in the basal ganglia between, PwHDt$_{otal}$ verus HC$_{total}$. Voxel-wise statistical parametric maps from group comparison of MPM modalities in the basal ganglia are presented on the used basal ganglia mask at 0, 0, 0 mm (MNI space) in axial and coronal views. Color bars show voxel-wise t-values, with higher absolute values indicating stronger group differences. A. Clusters with lower R1 values in PwHD$_{total}$ (in blue, T = 3.22, FWEc = 117, df = [1, 64]). B. Clusters with lower R2* values in PwHD$_{total}$ (in blue, T = 3.22, FWEc = 146, df = [1, 64]). C. Clusters with lower PD values in PwHD$_{total}$ (in blue, T = 3.22, FWEc = 111, df = [1, 64]). D. Clusters with lower MTsat values in PwHD$_{total}$ (in blue, T = 3.22, FWEc = 608, df [1, 64]). df: degrees of freedom. FWE: family-wise error. HC$_{total}$: healthy controls. L: left hemisphere. MPM: multiparametric mapping. MTsat: magnetization transfer saturation. PD: proton density. PwHD$_{total}$: premanifest and manifesting patients with Huntington's disease. R: right hemisphere. R1: longitudinal relaxation rate. R2*: effective transverse relaxation rate. T: t-statistic.

Table S6. Clusters with significant group-level VBM differences across MPM modalities in the basal ganglia, $PwHD_{total}$ versus HC_{total}.

	cluster level				*peak level*		*mm*	*mm*	*mm*
	$P_{FWE\text{-}corr}$	$q_{FRD\text{-}corr}$	K_E	P_{uncorr}	T	(Z_E)			
VBM – $PwHD_{total}$ < HC_{total}									
lCaudate	0.000	0.000	4188	0.000	5.27	4.78	-18	-16	27
rCaudate	0.000	0.000	3764	0.000	4.59	4.25	18	-3	28
R1 - $PwHD_{total}$ < HC_{total}									
rCaudate	0.000	0.002	447	0.000	4.07	3.82	10	5	9
lCaudate	0.041	0.382	117	0.020	3.63	3.45	-9	7	9
R2 - $PwHD_{total}$ < HC_{total}*									
rCaudate	0.000	0.000	1274	0.000	4.40	4.10	15	-1	22
lCaudate	0.017	0.124	146	0.008	3.86	3.64	-7	13	3
PD - $PwHD_{total}$ < HC_{total}									
rCaudate	0.040	0.566	111	0.018	3.86	3.64	13	-1	19
MTsat - $PwHD_{total}$ < HC_{total}									
lCaudate	0.000	0.000	1102	0.000	4.33	4.04	-8	9	6
rCaudate	0.000	0.000	608	0.000	4.14	3.88	19	-21	22

FDR_{corr}: false-discovery rate-corrected. FWE_{corr}: family-wise error-corrected. HC_{total}: healthy controls. K_E: cluster extent. lCaudate: left caudate nucleus. MPM: multiparametric mapping. MTsat: magnetization transfer saturation. PD: proton density. $PwHD_{total}$: premanifest and manifesting patients with Huntington's disease. R1: longitudinal relaxation rate. $R2^*$: effective transverse relaxation rate. rCaudate: right caudate nucleus. T: *t*-statistic. Uncorr: uncorrected. Z_E: equivalent z-score.

Table S7. Clusters with significant group-level differences across MPM modalities in whole-brain gray matter, $PwHD_{pre}$ versus HC_{pre}.

	cluster level				*peak level*				
	$P_{FWE-corr}$	$q_{FRD-corr}$	K_E	P_{uncorr}	*T*	(Z_E)	*mm*	*mm*	*mm*
R1 – $PwHD_{pre}$ < HC_{pre}									
lSFG	0.028	0.016	191	0.000	5.53	4.53	-3	-8	78
lMTG	0.031	0.016	188	0.000	3.95	3.50	16	39	-24
R2 - $PwHD_{pre}$ > HC_{pre}*									
lPOrG	0.000	0.000	392	0.000	4.96	4.19	-29	21	-11
R2 - $PwHD_{pre}$ < HC_{pre}*									
rMFG	0.000	0.000	324	0.000	5.26	4.37	29	44	15
rSPL	0.000	0.000	396	0.000	4.84	4.11	24	-39	70
MTsat – $PwHD_{pre}$ < HC_{pre}									
rSFG	0.001	0.001	321	0.000	5.82	4.70	25	-8	63
rPCgG	0.014	0.002	212	0.000	4.45	3.86	-67	-53	-12
VBM – $PwHD_{pre}$ < HC_{pre}									
CVL	0.000	0.000	1017	0.000	28.76	4.24	-4	-51	-33
rMOrG	0.007	0.004	559	0.000	23.26	3.90	6	34	-22

The HC_{pre} group comprises only healthy participants, and “pre” labeling refers to the matched $PwHD_{pre}$ group. CVL: cerebellar vermal lobules I-V. FDR_{corr}: false-discovery rate-corrected. FWE_{corr}: family-wise error-corrected. HC_{pre}: healthy controls. K_E: cluster extent. lMTG: left middle temporal gyrus. lPOrG: left posterior orbital gyrus. lSFG: left superior frontal gyrus. MPM: multiparametric mapping. MTsat: magnetization transfer saturation. $PwHD_{Pre}$: premanifest patients with Huntington’s disease. R1: longitudinal relaxation rate. $R2^*$: effective transverse relaxation rate. rMFG: right middle frontal gyrus. rMOrG: right middle orbital gyrus. rPCgG: right posterior cingulate gyrus. rSFG: right superior frontal gyrus. rSPL: right superior parietal lobule. T: *t*-statistic. Uncorr: uncorrected. Z_E: equivalent z-score.

Table S8. Clusters with group-level significant R1 differences in the whole-brain gray matter, $PwHD_{man}$ versus HC_{man}.

	cluster level				*peak level*		*mm*	*mm*	*mm*
	$P_{FWE\text{-}corr}$	$q_{FRD\text{-}corr}$	K_E	P_{uncorr}	T	(Z_E)			
$PwHD_{man} < HC_{man}$									
lSTG	0.000	0.000	823	0.000	10.65	5.71	-67	-6	-7
rITG	0.022	0.006	232	0.000	8.46	5.14	53	0	-48
lPoG	0.000	0.000	558	0.000	8.21	5.07	-44	-19	66
lMCgG	0.003	0.001	315	0.000	8.20	5.07	-13	10	32
lCaudate	0.000	0.000	873	0.000	6.94	4.65	-3	16	4
lCE	0.040	0.008	210	0.000	6.83	4.61	-5	-92	-35
rPoG	0.028	0.006	224	0.000	6.71	4.51	26	-30	75
rMFG	0.004	0.001	300	0.000	6.05	4.30	45	40	28
lMFC	0.000	0.000	547	0.000	5.73	4.17	-8	61	-8
lSMG	0.025	0.006	228	0.000	5.21	3.93	-49	-48	42
lIOG	0.000	0.000	486	0.000	4.77	3.71	-37	-94	-5

The HC_{man} group comprises only healthy participants, and "man" labeling refers to the matched $PwHD_{man}$ group. FDR_{corr}: false-discovery rate-corrected. FWE_{corr}: family-wise error-corrected. HC_{man}: healthy controls. K_E: cluster extent. lCaudate: left caudate nucleus. lCE: left cerebellum exterior. lIOG: left inferior occipital gyrus. lMCgG: left middle cingulate gyrus. lMFC: left middle frontal cortex. lPoG: left postcentral gyrus. lSMG: left supramarginal gyrus. lSTG: left superior temporal gyrus. $PwHD_{man}$: manifesting patients with Huntington's disease. R1: longitudinal relaxation rate. rITG: right inferior temporal gyrus. rMFG: right middle frontal gyrus. rPoG: right postcentral gyrus. T: *t*-statistic. Uncorr: uncorrected. Z_E: equivalent z-score.

Table S9. Clusters with significant group-level R2* differences in the whole-brain gray matter, $PwHD_{man}$ versus HC_{man}.

	cluster level				*peak level*		*mm*	*mm*	*mm*
	$P_{FWE\text{-}corr}$	$q_{FRD\text{-}corr}$	K_E	P_{uncorr}	*T*	(Z_E)			
$PwHD_{man}$ < HC_{man}									
lTMP	0.000	0.000	783	0.000	11.87	5.97	-62	8	-18
lMTG	0.030	0.003	209	0.000	10.07	5.57	-41	-53	6
lIOG	0.000	0.000	1176	0.000	9.14	5.34	-40	-92	5
rHip	0.001	0.000	362	0.000	8.69	5.21	36	-5	-24
lMCgG	0.002	0.000	305	0.000	8.10	5.04	-13	10	32
rSMG	0.000	0.000	415	0.000	7.81	4.95	60	-20	37
rAnG	0.000	0.000	1568	0.000	7.49	4.84	48	-60	49
lPoG	0.004	0.000	283	0.000	6.67	4.55	-44	-19	66
rPoG	0.000	0.000	468	0.000	6.60	4.52	26	-30	75
rPoG	0.000	0.000	1855	0.000	6.50	4.48	47	-15	55
lOrIFG	0.011	0.001	245	0.000	6.34	4.42	-46	43	-9
lMFG	0.001	0.000	353	0.000	6.13	4.34	-29	40	33
rPoG	0.000	0.000	554	0.000	6.06	4.31	14	-33	68
lSPL	0.000	0.000	373	0.000	5.84	4.22	-25	-58	41
rMOrG	0.000	0.000	546	0.000	5.71	4.16	20	27	-16
rCun	0.008	0.001	259	0.000	5.61	4.11	6	-78	35
lPCun	0.000	0.000	592	0.000	5.60	4.11	-12	-49	45
rFuG	0.033	0.003	205	0.000	5.51	4.07	40	-17	-23
lPCun	0.000	0.000	805	0.000	5.42	4.03	-17	-72	28
rSOG	0.000	0.000	589	0.000	5.26	3.95	30	-72	25
rCaudate	0.000	0.000	837	0.000	5.24	3.94	7	15	-1
rCO	0.000	0.000	1489	0.000	5.21	3.93	48	-15	16
lSTG	0.000	0.000	1320	0.000	5.09	3.87	-66	-45	11
rIOG	0.000	0.000	574	0.000	5.07	3.86	48	-75	8
rPO	0.009	0.001	251	0.000	4.99	3.82	50	-28	24
rMOG	0.007	0.001	263	0.000	4.99	3.82	37	-85	15
rSFG	0.022	0.002	220	0.000	4.86	3.76	14	14	56
rPO	0.002	0.000	316	0.000	4.84	3.75	57	-37	37
rPrG	0.000	0.000	483	0.000	4.81	3.73	22	-23	54
lPoG	0.017	0.002	229	0.000	4.77	3.71	-63	-9	32
rThal	0.004	0.000	286	0.000	4.69	3.67	6	-31	8
lPoG	0.003	0.000	300	0.000	4.37	3.49	-35	-25	46

The HC_{man} group comprises only healthy participants, and "man" labeling refers to the matched $PwHD_{man}$ group. FDR_{corr}: false-discovery rate-corrected. FWE_{corr}: family-wise error-corrected. HC_{man}: healthy controls. K_E: cluster extent. lIOG: left inferior occipital gyrus. lMCgG: left middle cingulate gyrus. lMFG: left middle frontal gyrus. lMTG: left middle temporal gyrus. lOrIFG: orbital part of the left inferior frontal gyrus. lPCun: left precuneus. lPoG: left postcentral gyrus. lSPL: left superior parietal lobule. lSTG: left superior temporal gyrus. lTMP: left temporal pole. $PwHD_{man}$: manifesting patients with Huntington's disease. $R2^*$: effective transverse relaxation rate. rAnG: right angular gyrus. rCaudate: right caudate nucleus. rCO: right central operculum. rCun: right cuneus. rFuG: right fusiform gyrus. rHip: right hippocampus. rIOG: right inferior occipital gyrus. rMOrG: right middle orbital gyrus. rPO: right parietal operculum. rPrG: right precentral gyrus. rPoG: right postcentral gyrus. rSFG: right superior frontal gyrus. rSMG: right supramarginal gyrus. rSOG: right superior occipital gyrus. rThal: right thalamus. T: *t*-statistic. Uncorr: uncorrected. Z_E: equivalent z-score.

Table S10. Clusters with significant group-level PD differences in the whole-brain gray matter, $PwHD_{man}$ versus HC_{man}.

	cluster level				*peak level*		*mm*	*mm*	*mm*
	$P_{FWE\text{-}corr}$	$q_{FRD\text{-}corr}$	K_E	P_{uncorr}	T	(Z_E)			
$PwHD_{man} < HC_{man}$									
lSTG	0.016	0.005	275	0.000	6.96	4.66	-64	6	-12
rCaudate	0.008	0.004	304	0.000	6.81	4.60	12	13	21
lCaudate	0.001	0.001	402	0.000	5.65	4.13	-9	2	20

The HC_{man} group comprises only healthy participants, and "man" labeling refers to the matched $PwHD_{man}$ group. FDR_{corr}: false-discovery rate-corrected. FWE_{corr}: family-wise error-corrected. HC_{man}: healthy controls. K_E: cluster extent. lCaudate: left caudate nucleus. lSTG: left superior temporal gyrus. PD: proton density. $PwHD_{man}$: manifesting patients with Huntington's disease. rCaudate: right caudate nucleus. T: *t*-statistic. Uncorr: uncorrected. Z_E: equivalent z-score.

Table S11. Clusters with significant group-level MTsat differences in the whole-brain gray matter, $PwHD_{man}$ versus HC_{man}.

	cluster level				*peak level*		*mm*	*mm*	*mm*
	$P_{FWE\text{-}corr}$	$q_{FRD\text{-}corr}$	K_E	P_{uncorr}	*T*	(Z_E)			
$PwHD_{man}$ < HC_{man}									
lSTG	0.016	0.004	282	0.041	10.72	5.73	-67	-6	-8
CVL	0.000	0.000	572	0.000	7.16	4.73	-3	-72	-17
lCE	0.000	0.000	764	0.000	6.93	4.65	-9	-94	-34
lCE	0.000	0.000	468	0.000	6.84	4.61	-22	-61	-16
lThal	0.000	0.000	8123	0.000	6.10	4.32	-7	-13	16
rHip	0.000	0.000	1182	0.000	5.19	3.92	34	-22	-12

The HC_{man} group comprises only healthy participants, and “man” labeling refers to the matched $PwHD_{man}$ group. CVL: cerebellar vermal lobules. FDR_{corr}: false-discovery rate-corrected. FWE_{corr}: family-wise error corrected. HC_{man}: healthy controls. K_E: cluster extent. lCE: left cerebellum exterior. lSTG: left superior temporal gyrus. lThal: left thalamus. MTsat: magnetization transfer saturation. $PwHD_{man}$: manifesting patients with Huntington’s disease. rHip: right hippocampus. T: *t*-statistic. Uncorr: uncorrected. Z_E: equivalent z-score.

Table S12. Clusters with significant group-level VBM differences in the whole-brain gray matter, $PwHD_{man}$ versus HC_{man}.

	cluster level				*peak level*		*mm*	*mm*	*mm*
	$P_{FWE\text{-}corr}$	$q_{FRD\text{-}corr}$	K_E	P_{uncorr}	T	(Z_E)			
$PwHD_{man} < HC_{man}$									
rFuG	0.000	0.000	113713	0.000	16.32	6.39	28	-38	-18
rPoG	0.000	0.000	5327	0.000	10.46	5.44	46	-22	48
rSPL	0.023	0.005	445	0.000	7.33	4.63	22	-54	66
rSFG	0.001	0.000	712	0.000	7.33	4.62	21	10	54
rMFG	0.023	0.005	445	0.000	6.45	4.32	27	22	36
lMFG	0.000	0.000	1063	0.000	6.42	4.31	-24	21	48

The HC_{man} group comprises only healthy participants, and "man" labeling refers to the matched $PwHD_{man}$ group. FDR_{corr}: false-discovery rate-corrected. FWE_{corr}: family-wise error-corrected. HC_{man}: healthy controls. K_E: cluster extent. lMFG: left middle frontal gyrus. $PwHD_{man}$: manifesting patients with Huntington's disease. rFuG: right fusiform gyrus. rMFG: right middle frontal gyrus. rPoG: right postcentral gyrus. rSFG: right superior frontal gyrus. rSPL: right superior parietal gyrus. T: *t*-statistic. Uncorr: uncorrected. VBM: volume-based morphometry. Z_E: equivalent z-score.

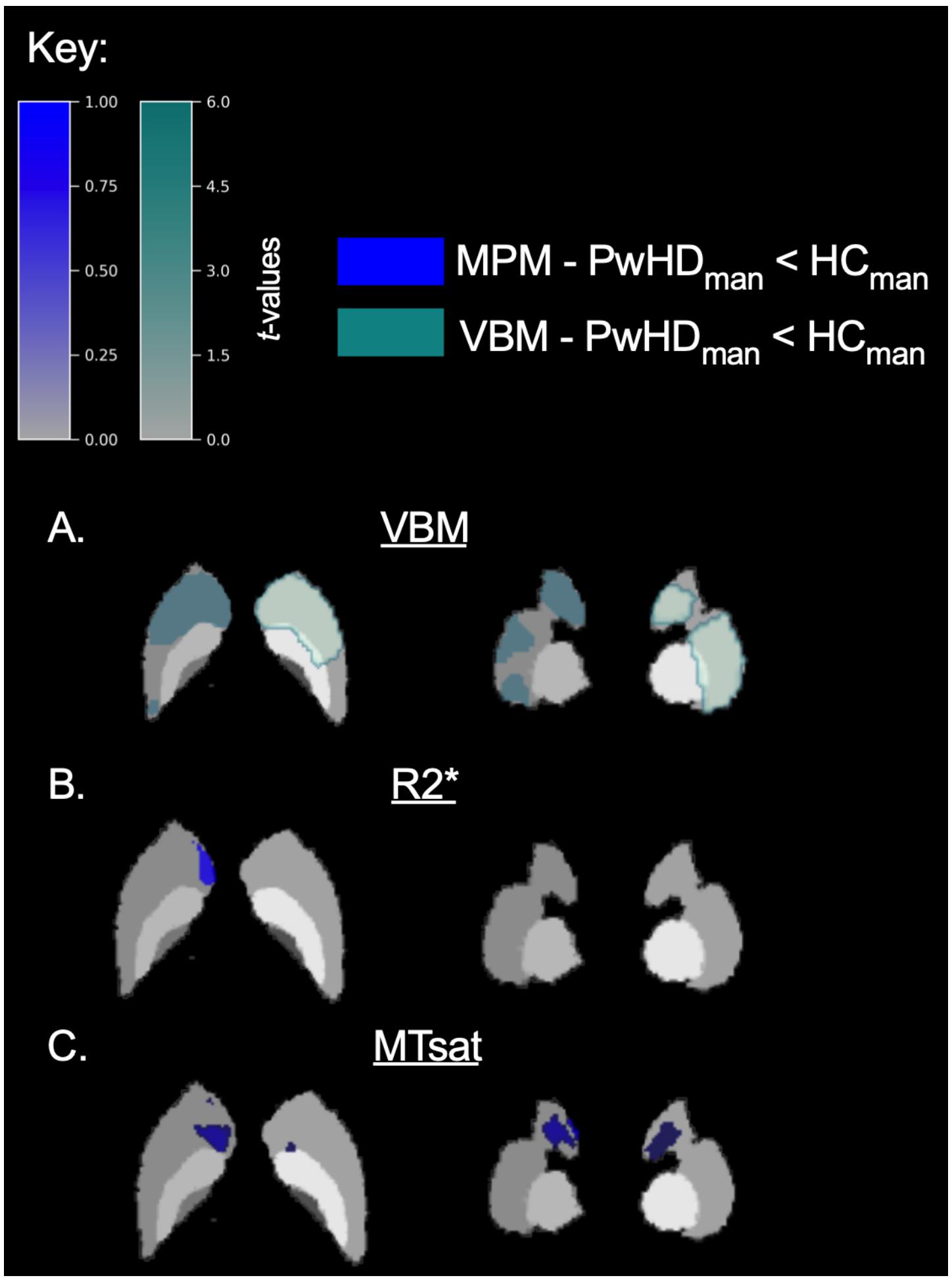

Figure S3. Group-level differences across MPM modalities in the basal ganglia, PwHD$_{man}$ versus HC$_{man}$. Voxel-wise statistical parametric maps from group comparison of MPM modalities in the basal ganglia are presented on the used basal ganglia mask at 0, 0, 0 mm (MNI space) in axial and coronal views. Color bars present voxel-wise t-values, with higher absolute values indicating stronger group differences. In panel A, the VBM results are presented, demonstrating clusters with lower t-values in PwHD$_{man}$ (in teal, T = 3.79, FWEc = 4226, df = [1, 14]). B. Clusters with lower R2* t-values in PwHD$_{man}$ (in blue, T = 3.69, FWEc = 343, df = [1, 16]). C. Clusters with lower MTsat t-values in PwHD$_{man}$ (T = 3.69, FWEc = 150, df = [1, 16]). The HC$_{man}$ group comprises only healthy participants, and "man" labelling refers to the matched PwHD$_{man}$ group. df: degrees of freedom. FWE: family-wise error. HC$_{man}$: healthy controls. L: left hemisphere. MPM: multiparametric mapping. MTsat: magnetization transfer saturation. PwHD$_{man}$: manifesting patients with Huntington's disease. R: right hemisphere. R2*: effective transverse relaxation rate. T: t-statistic. VBM: volume-based morphometry.

Table S13. Clusters with significant group-level differences across MPM modalities and VBM in the basal ganglia, $PwHD_{man}$ versus HC_{man}.

	cluster level				*peak level*		*mm*	*mm*	*mm*
	$P_{FWE\text{-}corr}$	$q_{FRD\text{-}corr}$	K_E	P_{uncorr}	*T*	(Z_E)			
R2 - $PwHD_{man}$ < HC_{man}*									
rCaudate	0.001	0.015	343	0.001	4.75	3.70	8	16	-1
MTsat – $PwHD_{man}$ < HC_{man}									
lCaudate	0.000	0.000	1480	0.000	6.06	4.31	-18	-23	20
rCaudate	0.000	0.000	1310	0.000	5.23	3.94	19	-21	22
rCaudate	0.038	0.124	150	0.023	4.60	3.62	11	-2	20
VBM – $PwHD_{man}$ < HC_{man}									
rPutamen	0.000	0.000	4296	0.000	5.37	.000	24	6	-12
lPutamen	0.000	0.000	4226	0.000	5.07	.000	-15	4	-15

The HC_{man} group comprises only healthy participants, and "man" labeling refers to the matched $PwHD_{man}$ group. FDR_{corr}: false-discovery rate-corrected. FWE_{corr}: family-wise error-corrected. HC_{man}: healthy controls. K_E: cluster extent. lCaudate: left caudate nucleus. lPutamen: left putamen. MPM: multiparametric mapping. MTsat: magnetization transfer saturation. $PwHD_{man}$: manifesting patients with Huntington's disease. $R2^*$: effective transverse relaxation rate. rCaudate: right caudate nucleus. rPutamen: right putamen. T: *t*-statistic. Uncorr: uncorrected. VBM: volume-based morphometry. Z_E: equivalent z-score.

Table S14. Cross-modal correlations between MPM modalities in the whole-brain gray matter clusters with significant group-level differences.

Anatomical location	Affected modalities		r/rho	p_{uncorr}	p_{FDR}
**$PwHD_{total}$ vs HC_{total}**					
lFuG	R1	R2*	0.953*	< 0.0001	< 0.0002
	R1	MTsat	0.836*		
	R2*	MTsat	0.892		
lMTG	R1	PD	0.966*	< 0.0001	< 0.0002
rThal	R1	R2*	0.955*	< 0.0001	< 0.0002
	R1	MTsat	0.707*		
	R2*	MTsat	0.691*		
lMOG	R1	R2*	0.551*	0.0035	0.0043
lCaudate	R1	R2*	0.690*	< 0.0001	< 0.0002
	R1	MTsat	0.910*		
	R2*	MTsat	0.753*		
rCaudate	R1	R2*	0.694*	< 0.0001	< 0.0002
	R1	MTsat	0.934*		
	R2*	MTsat	0.837*		
**$PwHD_{man}$ vs HC_{man}**					
lSTG	R1	R2*	0.660*	0.0438	0.0438
	R1	MTsat	0.672*	0.0390	0.0410
lPoG	R1	R2*	0.878*	0.0016	0.0021
lMCgG	R1	R2*	0.987*	0.0001	0.0002
rPoG	R1	R2*	0.927*	0.0003	0.0004
lIOG	R1	R2*	0.745*	0.0174	0.0203
rCaudare	R2*	MTsat	0.699	0.0243	0.0269

[*] according to Spearman's correlation. lCaudate: left caudate nucleus. lFuG: left fusiform gyrus. lIOG: left inferior occipital gyrus. lMCgG: left middle cingulate gyrus. lMOG: left middle occipital gyrus. lMTG: left middle temporal gyrus. lPoG: left postcentral gyrus. lSTG: left superior temporal gyrus. The HC_{man} group comprises only healthy participants, and “man” labeling refers to the matched $PwHD_{man}$ group. $HC_{total/man}$: healthy controls. MPM: multiparametric mapping. MTsat: magnetization transfer saturation. PD: proton density. p_{FDR}: false discovery rate-corrected p-value. $PwHD_{man}$: manifesting patients with Huntington’s disease. $PwHD_{total}$: premanifest and manifesting patients with Huntington’s disease. p_{uncorr}: uncorrected p-valued. r/rho: Pearson’s/Spearman’s correlation coefficient. R1: longitudinal relaxation rate. R2*: effective transverse relaxation rate. rCaudate: right caudate nucleus. rPoG: right postcentral gyrus. rThal: right thalamus.

Table S15. Associations between HD-related MPM alterations and clinical variables in PwHD$_{total}$.

MPM modality	Anatomical location	Clinical variable	rho	p_{uncorr}	p_{FDR}
R1	lCE	Disease duration	-0.644	0.0444	0.0473
		MoCA	0.597	0.0013	0.0040
		UHDRS	-0.596	0.0013	0.0040
	lCaudate	Disease duration	-0.704	0.0001	0.0005
		MoCA	0.429	0.0289	0.0359
		UHDRS	-0.774	< 0.0001	0.0003
	lIOG	MoCA	-0.530	0.0038	0.0093
		UHDRS	-0.591	0.0015	0.0044
	lMCgC	mHTT	0.636	0.0483	0.0492
	lMOG	Disease duration	-0.482	0.0126	0.0196
		MoCA	0.559	0.0030	0.0080
		UHDRS	-0.624	0.0007	0.0026
	lMTG	Disease duration	-0.436	0.0261	0.0341
		MoCA	0.454	0.0197	0.0267
	lPOrG	Disease duration	-0.548	0.0038	0.0093
		MoCA	0.471	0.051	0.0223
		UHDRS	-0.706	0.0001	0.0005
	rAOrG	MoCA	0.389	0.0494	0.0498
	rCaudate	Disease duration	-0.760	< 0.0001	0.0003
		MoCA	0.560	0.0029	0.0079
		UHDRS	-0.822	< 0.0001	0.0003
	rMOrG	MoCA	0.465	0.0166	0.0239
	rThalamus	Disease duration	-0.680	0.0001	0.0005
		UHDRS	-0.619	0.0007	0.0026
R2*	lCaudate	Disease duration	-0.756	< 0.0001	0.0003
		MoCA	0.477	0.0137	0.0210
		UHDRS	-0.799	< 0.0001	0.0003
	lIOG	MoCA	0.762	0.0104	0.0184
	lMCgG	Disease duration	-0.727	0.0287	0.0359
		MoCA	0.619	0.0118	0.0188
		UHDRS	-0.616	0.0286	0.0359
		mHTT	0.636	0.0483	0.0492
	lMOG	Disease duration	-0.727	< 0.0001	0.0003
		MoCA	0.619	0.0070	0.0144
		UHDRS	-0.616	0.0008	0.0029
	lOrIFG	MoCA	0.646	0.0435	0.0473
		UHDRS	-0.671	0.0336	0.0397
	lPCun	MoCA	0.884	0.0069	0.0144
		UHDRS	-0.790	0.0007	0.0026
	lPoG	Disease duration	-0.519	0.0049	0.0110
		UHDRS	-0.608	0.0009	0.0031
	lPrG	Disease duration	-0.472	0.0149	0.0223
		UHDRS	-0.596	0.0013	0.0040
	lSFG	Disease duration	-0.427	0.0419	0.0468
		UHDRS	-0.402	0.0297	0.0362
	lSMG	Disease duration	-0.470	0.0153	0.0223
		MoCA	0.532	0.0051	0.0111
		UHDRS	-0.641	0.0004	0.006
		mHTT	0.397	0.0387	0.0445
	lSTG	Disease duration	-0.405	0.0401	0.0457

		MoCA	0.513	0.0214	0.0286
	rAnG	MoCA	0.585	0.0017	0.0049
		UHDRS	-0.722	< 0.0001	0.0003
	rCO	MoCA	0.671	0.0338	0.0397
		UHDRS	-0.638	0.0472	0.0489
	rCaudate	Disease duration	-0.733	< 0.0001	0.0003
		MoCA	0.497	0.0099	0.0183
		UHDRS	-0.748	< 0.0001	0.0003
	rFuG	MoCA	0.756	0.0114	0.0188
		UHDRS	-0.764	0.0102	0.0183
	rIOG	MoCA	0.004	0.0040	0.0096
	rMFG	Disease duration	-0.487	0.0116	0.0188
		UHDRS	-0.397	0.0444	0.0473
	rMOG	MoCA	0.927	0.0001	0.0005
		UHDRS	-0.866	0.0012	0.0040
	rPoG	Disease duration	-0.424	0.0311	0.0373
		MoCA	0.811	0.0083	0.0167
		UHDRS	-0.662	0.0051	0.0111
	rPrG	Disease duration	-0.500	0.0093	0.0175
		MoCA	0.395	0.0044	0.0103
		UHDRS	-0.653	0.0003	0.0013
		mHTT	0.653	0.0372	0.0432
	rSFG	Disease duration	-0.487	0.0116	0.0188
		UHDRS	-0.397	0.0444	0.0473
	rSOG	MoCA	0.695	0.0256	0.0338
		UHDRS	-0.723	0.0183	0.0257
	rSTG	Disease duration	-0.473	0.0147	0.0222
		MoCA	0.503	0.0088	0.0169
		UHDRS	-0.595	0.0014	0.0042
		mHTT	0.394	0.0466	0.0487
	rThalamus	Disease duration	-0.664	0.0002	0.0009
		MoCA	0.765	0.0100	0.0183
		UHDRS	-0.580	0.0019	0.0053
PD	lCaudate	Disease duration	-0.659	< 0.0001	0.0003
		UHDRS	-0.712	< 0.0001	0.0003
	lMTG	MoCA	0.428	0.0290	0.0359
	rCaudate	Disease duration	-0.727	< 0.0001	0.0003
		MoCA	0.458	0.0187	0.0259
		UHDRS	-0.758	< 0.0001	0.0003
MTsat	lCaudate	Disease duration	-0.818	<0.0001	0.0003
		MoCA	0.489	0.0112	0.0188
		UHDRS	-0.750	< 0.0001	0.0003
	lMFG	UHDRS	-0.403	0.0415	0.0468
	lPOrG	Disease duration	-0.506	0.0084	0.0167
		UHDRS	-0.537	0.0047	0.0108
	lSMG	Disease duration	-0.555	0.0033	0.0086
		MoCA	0.488	0.0114	0.0188
		UHDRS	-0.487	0.0116	0.0188
	lSTG	Disease duration	-0.395	0.0456	0.0481
		MoCA	0.399	0.0434	0.0473
		UHDRS	-0.388	0.0499	0.0499
		mHTT	0.685	0.0287	0.0359
	rCaudate	Disease duration	-0.714	< 0.0001	0.0003
		MoCA	0.426	0.0299	0.0362

		UHDRS	-0.780	< 0.0001	0.0003
	rHip	Disease duration	-0.728	< 0.0001	0.0003
		MoCA	0.461	0.0178	0.0253
		UHDRS	-0.763	< 0.0001	0.0003
	rMTG	Disease duration	-0.529	0.0054	0.0115
		UHDRS	-0.550	0.0036	0.0092
	rSTG	Disease duration	-0.485	0.0121	0.0191
		UHDRS	-0.456	0.0192	0.0263
	rThalamus	Disease duration	-0.780	< 0.0001	0.0003
		MoCA	0.504	0.0087	0.0169
		UHDRS	-0.785	< 0.0001	0.0003

lCaudate: left caudate nucleus. lCE: left cerebellum exterior. lFuG: left fusiform gyrus. lIOG: left inferior occipital gyrus. lMCgG: left middle cingulate gyrus. lMOG: left middle occipital gyrus. lMTG: left middle temporal gyrus. lOrIFG: left inferior frontal gyrus pars orbitalis. lPCun: left precuneus. lPoG: left postcentral gyrus. lPOrG: left posterior orbital gyrus. lPrG: left precentral gyrus. lSFG: left superior frontal gyrus. lSMG: left supramarginal gyrus. lSTG: left superior temporal gyrus. MPM: multiparametric mapping. MTsat: magnetization transfer saturation. PD: proton density. p_{FDR}: false discovery rate-corrected p-value. $PwHD_{man}$: manifesting patients with Huntington's disease. p_{uncorr}: uncorrected p-valued. rho: Spearman's correlation coefficient. R1: longitudinal relaxation rate. R2*: effective transverse relaxation rate. rAnG: right angular gyrus. rAOrG: right anterior orbital gyrus. rCaudate: right caudate nucleus. rCO: right central operculum. rFuG: right fusiform gyrus. rHip: right hippocampus. rIOG: right inferior occipital gyrus. rMFG: right middle frontal gyrus. rMOG: right middle occipital gyrus. rMOrG: right medial orbital gyrus. rMTG: right middle temporal gyrus. rPoG: right postcentral gyrus. rPrG: right precentral gyrus. rSFG: right superior frontal gyrus. rSOG: right superior occipital gyrus. rSTG: right superior temporal gyrus. rThalamus: right thalamus.

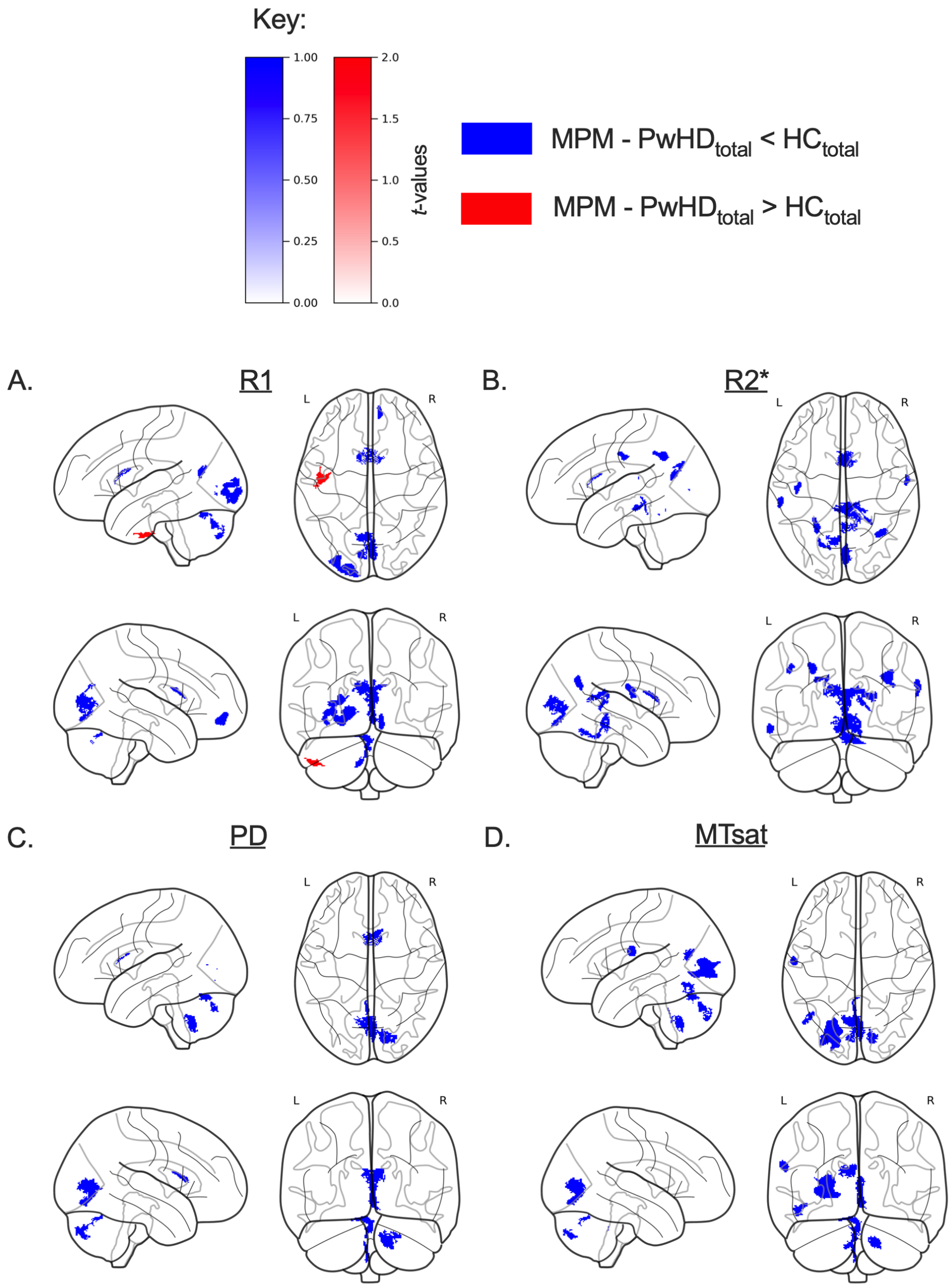

Figure S4. Group-level differences across MPM modalities in the whole-brain white matter, $PwHD_{total}$ versus HC_{total}. Voxel-wise statistical parametric maps from group comparison of MPM modalities are presented on a standard brain template in sagittal, axial, and coronal views. Color bars present voxel-wise t-values, with higher absolute values indicating stronger group differences. A. Clusters with lower R1 values in $PwHD_{total}$ (in blue) and clusters with higher R1 values in $PwHD_{total}$ (in red, T = 3.22, FWEc = 151, df = [1, 64]). B. Clusters with lower R2* values in $PwHD_{total}$ (in blue, T = 3.22, FWEc = 150, df = [1, 64]). C. Clusters with lower PD values in $PwHD_{total}$ (in blue, T = 3.22, FWEc = 186, df = [1, 64]). D. Clusters with lower MTsat values in $PwHD_{total}$ (in blue, T = 3.22, FWEc = 185, df = [1,64]). df: degrees of freedom. FWE: family-wise error. HC_{total}: healthy controls. L: left hemisphere. MPM: multiparametric mapping. MTsat: magnetization transfer saturation. PD: proton density. PwHDtotal: premanifest and manifesting patients with Huntington's disease. R: right hemisphere. R1: longitudinal relaxation rate. R2*: effective transverse relaxation rate. T: t-statistic.

Table S16. Clusters with significant group-level differences across MPM modalities in the whole-brain white matter, PwHD$_{total}$ versus HC$_{total}$.

	cluster level				*peak level*		*mm*	*mm*	*mm*
	$P_{FWE-corr}$	$q_{FRD-corr}$	K_E	P_{uncorr}	T	(Z_E)			
R1 - PwHD$_{total}$ > HC$_{total}$									
lUF	0.001	0.000	254	0.000	6.20	5.47	-42	-8	-43
R1 – PwHD$_{total}$ < HC$_{total}$									
lC	0.018	0.004	167	0.000	5.51	4.96	-6	-65	18
MCP	0.033	0.007	151	0.000	5.24	4.77	-7	-66	-21
sCC	0.000	0.000	651	0.000	4.99	4.56	3	-75	4
MCP	0.016	0.004	170	0.000	4.65	4.30	-3	-75	-33
lPTR	0.000	0.000	715	0.000	4.50	4.18	-31	-93	7
bCC	0.002	0.001	223	0.000	4.19	3.92	3	6	19
lPTR	0.000	0.000	860	0.000	3.94	3.72	-14	-100	-1
rACR	0.000	0.000	294	0.000	3.85	3.64	8	52	-11
R2 – PwHD$_{total}$ < HC$_{total}$*									
lACR	0.004	0.001	196	0.000	5.32	4.82	-5	-66	19
rSCP	0.000	0.000	717	0.000	5.30	4.81	9	-35	-8
lACR	0.000	0.000	623	0.000	5.00	4.58	3	-75	4
bCC	0.000	0.000	279	0.000	4.41	4.10	3	6	19
lSS	0.019	0.004	155	0.000	4.37	4.07	-60	-28	-13
sCC	0.000	0.000	293	0.000	4.23	3.96	18	-36	9
lPTR	0.023	0.004	150	0.000	4.21	3.94	-14	-71	28
rSLF	0.001	0.000	233	0.000	4.19	3.92	64	-7	22
lPCR	0.000	0.000	376	0.000	3.92	3.69	-24	-54	37
lSLF	0.010	0.002	171	0.000	3.75	3.55	-43	-16	36
rSLF	0.000	0.000	378	0.000	3.70	3.51	37	-58	31
PD – PwHD$_{total}$ < HC$_{total}$									
sCC	0.000	0.000	744	0.000	5.89	5.25	3	-75	4
lACR	0.007	0.002	186	0.000	5.58	5.02	-4	-65	19
lICP	0.000	0.000	317	0.000	5.40	4.88	-2	-67	-23
lSCP	0.006	0.002	191	0.000	4.91	4.51	-3	-55	-44
MCP	0.000	0.000	366	0.000	4.78	4.40	16	-81	-42
bCC	0.002	0.001	214	0.000	4.05	3.81	3	6	19
MTsat – PwHD$_{total}$ < HC$_{total}$									
sCC	0.000	0.000	719	0.000	5.67	5.08	3	-75	4
lICP	0.000	0.000	398	0.000	5.61	5.04	-7	-66	-21
lC	0.002	0.001	227	0.000	5.60	5.03	-7	-64	17
lSS	0.000	0.000	292	0.000	4.87	4.48	-61	-9	21
lICP	0.008	0.002	189	0.000	4.82	4.43	-3	-53	-44
lPTR	0.010	0.002	185	0.000	4.65	4.30	-48	-66	-13
MCP	0.008	0.002	191	0.000	4.49	4.17	12	-81	-40
lPTR	0.000	0.000	3041	0.000	4.30	4.01	-24	-82	9

bCC: body of corpus callosum. FDR$_{corr}$: false-discovery rate-corrected. FWE$_{corr}$: family-wise error corrected. HC$_{total}$: healthy controls. K$_E$: cluster extent. lACR: left anterior corona radiata. lC: left cingulum. lICP: left inferior cerebellar peduncle. lPCR: left posterior corona radiata. lPTR: left posterior thalamic radiation. lSCP: left superior cerebella peduncle. lSLF: left superior longitudinal fasciculus. lSS: left sagittal stratum. lUF: left uncinate fasciculus. MCP: middle cerebellar peduncle. MPM: multiparametric mapping. MTsat: magnetization transfer saturation. PD: proton density. PwHD$_{total}$: premanifest and manifesting patients with

Huntington's disease. R1: longitudinal relaxation rate. $R2^*$: effective transverse relaxation rate. rACR: right anterior corona radiata. rSCP: right superior cerebellar peduncle. rSLF: right superior longitudinal fasciculus. sCC: splenium of corpus callosum. T: *t*-statistic. Uncorr: uncorrected. Z_E: equivalent z-score.

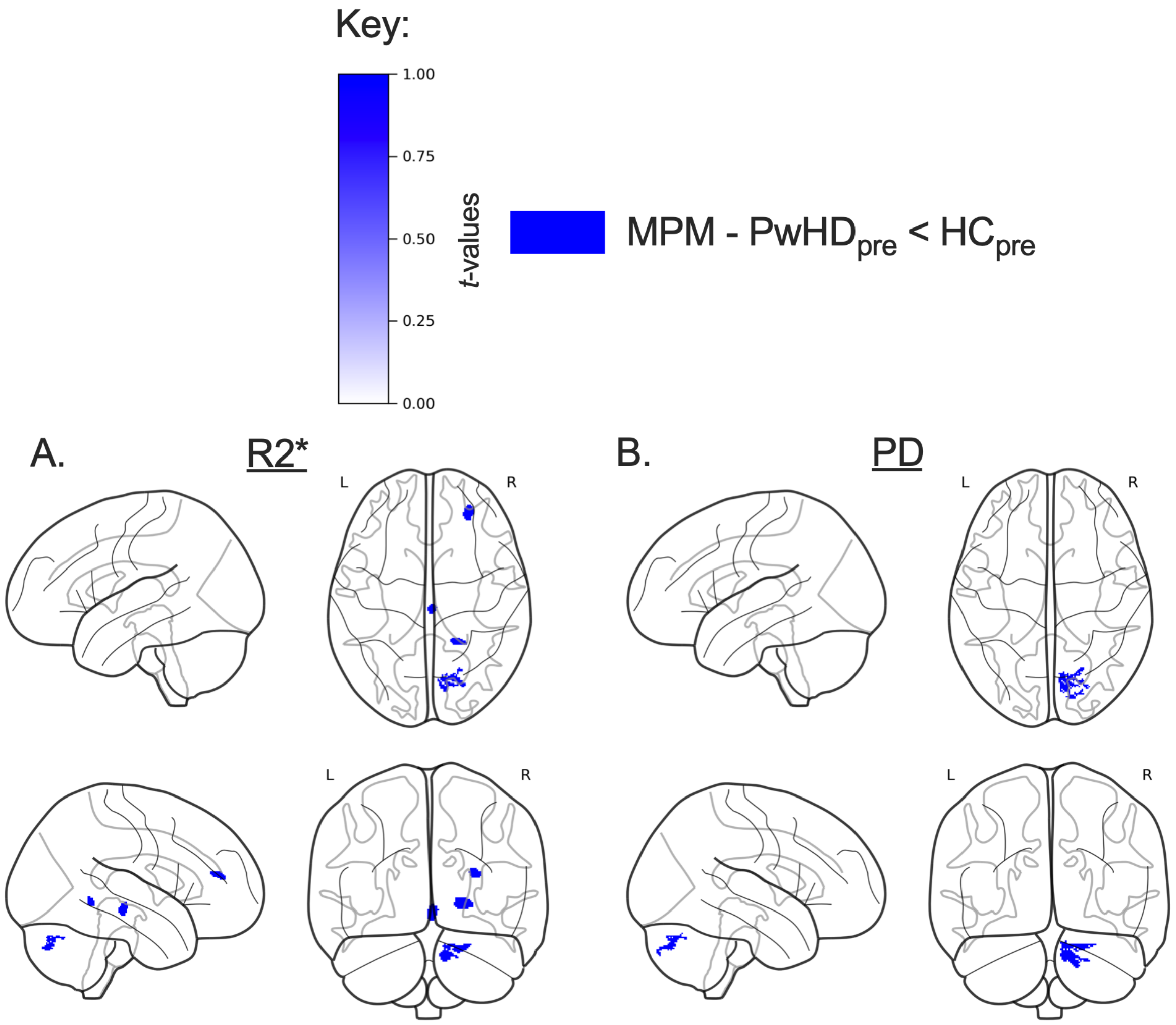

Figure S5. Group-level differences across MPM modalities in the whole-brain white matter, $PwHD_{pre}$ versus HC_{pre}. Voxel-wise statistical parametric maps from group comparison of MPM modalities are presented on a standard brain template in sagittal, axial, and coronal views. Color bars present voxel-wise t-values, with higher absolute values indicating stronger group differences. The HC_{pre} group comprises only healthy participants, and "pre" labelling refers to the matched $PwHD_{pre}$ group. A. Clusters with lower R2* values in $PwHD_{pre}$ (in blue, T = 3.69, FWEc = 207, df = [1, 16]). Clusters with lower PD values in $PwHD_{pre}$ (in blue, T = 3.69, FWEc = 287, df = [1, 16]). df: degrees of freedom. FWE: family-wise error. HC_{pre}: healthy controls. L: left hemisphere. MPM: multiparametric mapping. PD: proton density. $PwHD_{pre}$: premanifest patients with Huntington's disease. R: right hemisphere. R2*: effective transverse relaxation rate. T: t-statistic.

Table S17. Clusters with significant group-level differences across MPM modalities in the whole-brain white matter, $PwHD_{pre}$ and HC_{pre}.

	cluster level				*peak level*		*mm*	*mm*	*mm*
	$P_{FWE\text{-}corr}$	$q_{FRD\text{-}corr}$	K_E	P_{uncorr}	T	(Z_E)			
R2 - $PwHD_{pre} < HC_{pre}$*									
MCP	0.002	0.001	238	0.000	4.89	4.14	11	-75	-29
rC	0.007	0.001	206	0.000	4.75	4.05	19	-47	-2
rACR	0.005	0.001	216	0.000	4.07	3.59	28	44	17
rCP	0.008	0.001	201	0.000	3.92	3.48	2	-24	-7
PD - $PwHD_{pre} < HC_{pre}$									
MCP	0.000	0.000	311	0.000	5.09	4.27	15	-73	-28

The HC_{pre} group comprises only healthy participants, and "pre" labeling refers to the matched $PwHD_{pre}$ group. FDR_{corr}: false-discovery rate-corrected. FWE_{corr}: family-wise error corrected. HC_{pre}: healthy controls. K_E: cluster extent. MPM: multiparametric mapping. MCP: middle cerebellar peduncle. PD: proton density. $PwHD_{pre}$: premanifest patients with Huntington's disease. $R2^*$: effective transverse relaxation rate. rACR: right anterior corona radiata. rC: right cingulum. rCP: right cerebral peduncle. T: *t*-statistic. Uncorr: uncorrected. Z_E: equivalent z-score.

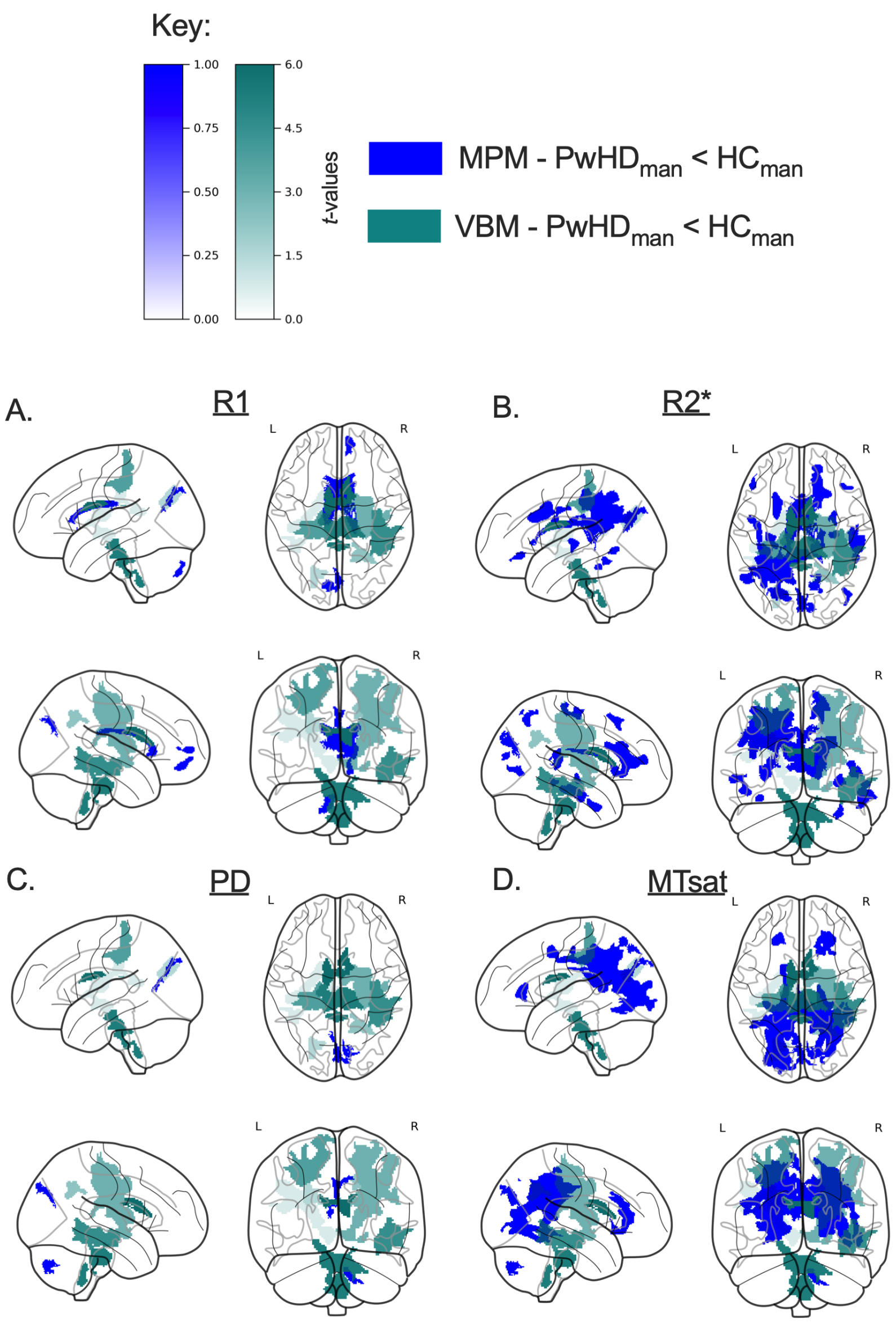

Figure S6. Group-level differences across MPM modalities in the whole-brain white matter, versus $PwHD_{man}$ and HC_{man}. Voxel-wise statistical parametric maps from group comparison of MPM modalities are presented on a standard brain template in sagittal, axial, and coronal views. Color bars present voxel-wise t-values, with higher absolute values indicating stronger group differences. The maps are overlaid with VBM-derived clusters, presenting lower t-values in PwHD (in teal, T = 3.79, FWEc = 419, df = [1, 14]. A. Clusters with lower R1 values in $PwHD_{man}$ (in blue, T = 3.69, FWEc = 252, df = [1, 16]). B. Clusters with lower R2* values in $PwHD_{man}$ (in blue, T = 3.69, FWEc = 207, df = [1, 16]). C. Clusters with lower PD values in $PwHD_{man}$ (in blue, T = 3.69, FWEc = 287, df = [1, 16]). D. Clusters with lower MTsat values in $PwHD_{man}$ (in blue, T = 3.69, FWEc = 245, df [1, 16]). The HC_{man} group comprises only healthy participants, and "man" labeling refers to the matched $PwHD_{man}$ group. df: degrees of freedom. FWE: family-wise error. HC_{man}: healthy controls. L: left hemisphere. MPM: multiparametric mapping. MTsat: magnetization transfer saturation. PD: proton density. $PwHD_{man}$: manifesting patients with Huntington's disease. R: right hemisphere. R1: longitudinal relaxation rate. R2*: effective transverse relaxation rate. T: t-statistic. VBM: volume-based morphometry.

Table S18. Clusters with significant group-level differences across MPM modalities in the whole-brain white matter, PwHD$_{man}$ versus HC$_{man}$.

	cluster level				*peak level*		*mm*	*mm*	*mm*
	$P_{FWE\text{-}corr}$	$q_{FRD\text{-}corr}$	K_E	P_{uncorr}	*T*	(Z_E)			
R1 – PwHD$_{man}$ < HC$_{man}$									
sCC	0.000	0.000	439	0.000	21.58	7.30	-1	-76	33
MCP	0.015	0.003	252	0.000	8.17	5.06	-7	-84	-40
gCC	0.000	0.000	2456	0.000	6.56	4.51	11	24	5
rACR	0.002	0.001	329	0.000	4.76	3.70	8	49	-11
rACR	0.001	0.001	356	0.000	4.28	3.44	10	58	5
R2 - PwHD$_{man}$ < HC$_{man}$*									
sCC	0.000	0.000	876	0.000	57.4	Inf	-2	-74	29
rSLF	0.000	0.000	21947	0.000	8.99	5.30	35	-42	37
rEC	0.003	0.000	289	0.000	7.35	4.79	34	-14	-5
lACR	0.000	0.000	15862	0.000	6.97	4.66	-13	-52	47
SS	0.000	0.000	1675	0.000	6.81	4.60	51	-16	-17
sCC	0.000	0.000	615	0.000	6.45	4.46	17	-35	10
lSLF	0.000	0.000	436	0.000	6.29	4.40	-57	-58	-1
rUF	0.000	0.000	363	0.000	6.26	4.39	32	-3	-27
gCC	0.000	0.000	6730	0.000	6.16	4.35	13	24	0
rPCR	0.000	0.000	502	0.000	5.80	4.20	19	-69	61
lACR	0.000	0.000	1968	0.000	5.59	4.10	-23	10	39
rPTR	0.000	0.000	419	0.000	5.11	3.88	46	-79	6
lSLF	0.000	0.000	668	0.000	5.09	3.87	-42	-5	38
lrIC	0.000	0.000	632	0.000	5.05	3.85	-28	-19	5
rSCR	0.000	0.000	852	0.000	4.89	3.77	19	-15	62
rPTR	0.019	0.001	219	0.000	4.81	3.73	8	-83	20
rACR	0.000	0.000	511	0.000	4.78	3.71	16	22	56
SS	0.026	0.002	207	0.000	4.72	3.68	-34	-42	-18
lEC	0.024	0.002	210	0.000	4.71	3.68	-48	36	-8
rACR	0.006	0.001	259	0.000	4.52	3.58	41	34	11
lACR	0.001	0.000	336	0.000	4.51	3.57	-47	-49	-12
SS	0.012	0.001	235	0.000	4.30	3.45	54	-37	-4
lSLF	0.014	0.001	230	0.000	4.12	3.35	-41	-17	28
lPCR	0.023	0.002	211	0.000	4.02	3.29	-23	-26	24
PD – PwHD$_{man}$ < HC$_{man}$									
lC	0.000	0.000	570	0.000	8.80	5.24	0	-74	26
MCP	0.006	0.003	287	0.000	6.47	4.47	12	-78	-43
MTsat – PwHD$_{man}$ < HC$_{man}$									
lPCR	0.003	0.001	307	0.000	23.57	7.49	-17	-80	50
lC	0.000	0.000	710	0.000	9.28	5.37	0	-74	26
sCC	0.000	0.000	45262	0.000	8.28	5.09	7	-40	19
sCC	0.000	0.000	920	0.000	6.42	4.45	25	-50	-1
rPTR	0.006	0.001	285	0.000	6.20	4.36	17	-80	28
lACR	0.000	0.000	918	0.000	5.91	4.24	-18	29	4
gCC	0.000	0.000	3016	0.000	5.83	4.21	17	30	4
gCC	0.007	0.001	279	0.000	5.38	4.01	5	19	11
MCP	0.003	0.001	317	0.000	5.34	3.99	13	-80	-42
SS	0.000	0.000	1014	0.000	4.77	3.71	47	-45	-7

The HC$_{man}$ group comprises only healthy participants, and “man” labeling refers to the matched PwHD$_{man}$ group. gCC: genu of corpus callosum. FDR$_{corr}$: false-discovery rate-corrected. FWE$_{corr}$: family-wise error corrected. HC$_{man}$: healthy controls. K$_E$: cluster extent.

lACR: left anterior corona radiata. lC: left cingulum. lEC: left external capsule. lPCR: left posterior corona radiata. lrIC: left retrolenticular part of the internal capsule. lSLF: left superior longitudinal fasciculus. MCP: middle cerebellar peduncle. MPM: multiparametric mapping. MTsat: magnetization transfer saturation. PD: proton density. $PwHD_{man}$: manifesting patients with Huntington’s disease. R1: longitudinal relaxation rate. $R2^*$: effective transverse relaxation rate. rACR: right anterior corona radiata. rEC: right external capsule. rPCR: right posterior corona radiata. rPTR: right posterior thalamic radiation. rSCR: right superior corona radiata. rSLF: right superior longitudinal fasciculus. rUF: right uncinate fasciculus. sCC: splenium of corpus callosum. SS: sagittal stratum. T: *t*-statistic. Uncorr: uncorrected. Z_E: equivalent z-score.

Table S19. Clusters with significant group-level differences across VBM in the whole-brain white matter, $PwHD_{man}$ versus HC_{man}.

	cluster level				peak level		mm	mm	mm
	$P_{FWE-corr}$	$q_{FRD-corr}$	K_E	P_{uncorr}	T	(Z_E)			
$PwHD_{man}$ < HC_{man}									
lPCR	0.011	0.003	556	0.000	7.52	4.69	-24	-81	32
rCP	0.000	0.000	6424	0.000	7.03	4.53	22	-4	-9
lCP	0.000	0.000	2223	0.000	6.43	4.32	-8	-14	-3
SS	0.000	0.000	2409	0.000	6.26	4.25	36	-32	-10
rSCR	0.016	0.004	519	0.000	6.16	4.22	24	18	48
lC	0.000	0.000	2479	0.000	6.11	4.20	0	-46	-52
lSCR	0.000	0.000	1795	0.000	6.02	4.16	-9	-32	60
rPCR	0.015	0.004	522	0.000	5.65	4.01	15	-56	39
bCC	0.044	0.009	419	0.001	5.21	3.82	10	9	21

The HC_{man} group comprises only healthy participants, and "man" labeling refers to the matched $PwHD_{man}$ group. bCC: body of corpus callosum. FDR_{corr}: false-discovery rate-corrected. FWE_{corr}: family-wise error corrected. HC_{man}: healthy controls. K_E: cluster extent. lC: left cingulum. lCP: left cerebral peduncle. lPCR: left posterior corona radiata. lSCR: left superior corona radiata. $PwHD_{man}$: manifesting patients with Huntington's disease. rCP: right cerebral peduncle. rPCR: right posterior corona radiata. rSCR: right superior corona radiata. SS: sagittal singulum. T: *t*-statistic. Uncorr: uncorrected. VBM: volume-based morphometry. Z_E: equivalent z-score.

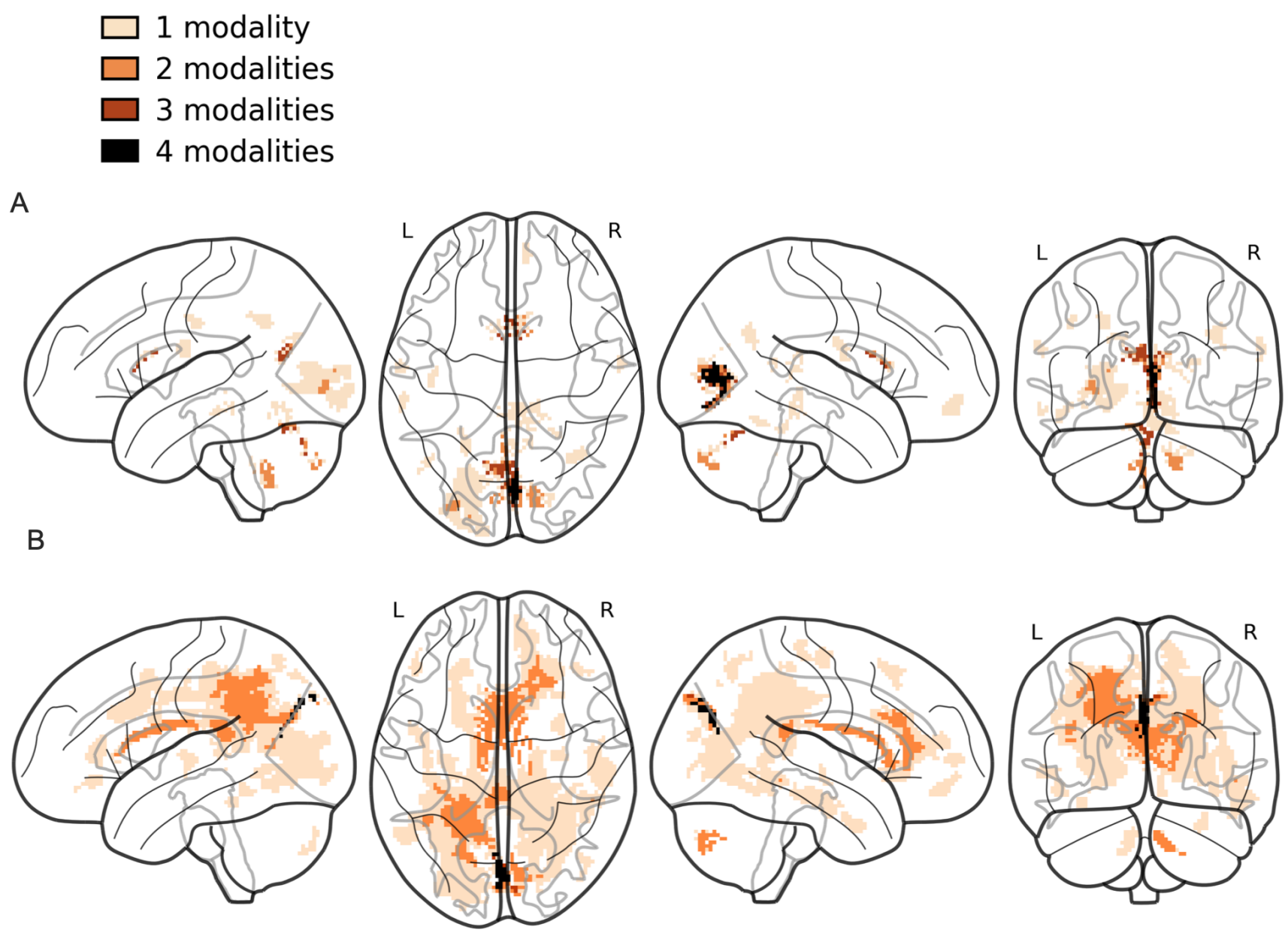

Figure S7. Venn-encoded whole-brain white matter overlap of MPM-derived statistical parametric maps. The figure presents overlaid maps of group differences across MPM modalities, color-coded by the number of cross-modal overlaps. A. Overlap of R1, R2*, PD, and MTsat differences between $PwHD_{total}$ and HC_{total}. B. Overlap of R1, R2*, PD, and MTsat differences between $PwHD_{man}$ and HC_{man}. The HC_{man} group comprises only healthy participants, and "man" labeling refers to the matched $PwHD_{man}$ group. $HC_{total/man}$: healthy controls. L: left hemisphere. MPM: multiparametric mapping. MTsat: magnetization transfer saturation. PD: proton density. $PwHD_{man}$: manifesting patients with Huntington's disease. $PwHD_{total}$: premanifest and manifesting patients with Huntington's disease. R: right hemisphere. R1: longitudinal relaxation rate. R2*: effective transverse relaxation rate.
